# Processing of predicted substrates of fungal Kex2 proteinases from *Candida albicans*, *C. glabrata*, *Saccharomyces cerevisiae *and *Pichia pastoris*

**DOI:** 10.1186/1471-2180-8-116

**Published:** 2008-07-14

**Authors:** Oliver Bader, Yannick Krauke, Bernhard Hube

**Affiliations:** 1 FG16, Robert Koch-Institut, Nordufer 20, D-13353 Berlin, Germany; 2 Institut für Medizinische Mikrobiologie, Universität Göttingen, Kreuzbergring 57, D-37075 Göttingen, Germany; 3 Dept. Membrane Transport, Institute of Physiology AS CR v.v.i., Videnska 1083, 142 20 Prague 4, Czech Republic; 4 Department of Microbial Pathogenicity, Leibniz Institute for Natural Product Research and Infection Biology – Hans Knoell Institute, Beutenbergstrasse 11a, D-07745 Jena, and Friedrich-Schiller-University Jena, Germany

## Abstract

**Background:**

Kexin-like proteinases are a subfamily of the subtilisin-like serine proteinases with multiple regulatory functions in eukaryotes. In the yeast *Saccharomyces cerevisiae *the Kex2 protein is biochemically well investigated, however, with the exception of a few well known proteins such as the α-pheromone precursors, killer toxin precursors and aspartic proteinase propeptides, very few substrates are known. Fungal *kex2 *deletion mutants display pleiotropic phenotypes that are thought to result from the failure to proteolytically activate such substrates.

**Results:**

In this study we have aimed at providing an improved assembly of Kex2 target proteins to explain the phenotypes observed in fungal *kex2 *deletion mutants by *in vitro *digestion of recombinant substrates from *Candida albicans *and *C. glabrata*. We identified CaEce1, CA0365, one member of the Pry protein family and CaOps4-homolog proteins as novel Kex2 substrates.

**Conclusion:**

Statistical analysis of the cleavage sites revealed extended subsite recognition of negatively charged residues in the P1', P2' and P4' positions, which is also reflected in construction of the respective binding pockets in the ScKex2 enzyme. Additionally, we provide evidence for the existence of structural constrains in potential substrates prohibiting proteolysis. Furthermore, by using purified Kex2 proteinases from *S. cerevisiae*, *P. pastoris*, *C. albicans *and *C. glabrata*, we show that while the substrate specificity is generally conserved between organisms, the proteinases are still distinct from each other and are likely to have additional unique substrate recognition.

## Background

Site specific proteolysis is a common feature in protein maturation and plays a crucial role in activation of many enzymes and in the generation of peptide hormones. In the late secretory pathway of eukaryotic cells this mechanism is mainly mediated by kexin-like proteinases, a subfamily of the subtilisin-like serine proteinases. Multicellular eukaryotes possess a large family of these regulatory proteinases, termed prohormone or proprotein convertases. While in mammals this family consists of at least seven members with tissue-specific expression patterns (most recently reviewed in [1]), fungi harbour only a single gene coding for a subtilisin-like serine proteinase with this activity. Originally identified in *kex2 *mutants of *Saccharomyces cerevisiae *lacking the ability to process the virally encoded killer toxin (killer expression) [2] the fungal Kex2 protein has since been implicated in several other proteolytic activation events, e.g. pheromone maturation at lysine-arginine motifs [3]. The *S. cerevisiae *Kex2 protein has been the target of substantial biochemical [4-6] and crystallographic (reviewed in [7]) research. Apart from *S. cerevisiae*, a diverse spectrum of phenotypic descriptions has been published for a range of *kex2 *deletion mutants from other yeasts, such as *Candida albicans *[8,9], *C. glabrata *[10], *Pichia pastoris *[11], *Schizosaccharomyces pombe *[12], or *Yarrowia lipolytica *[13] and moulds such as *Aspergillus niger *[14], *A. oryzae *[15] or *Trichoderma reesei *[16]. The phenotypes of these deletion mutants include morphological changes that are thought to result from the lack of activity from cell-wall modifying enzymes, reduced virulence in the case of *C. albicans *[9], hypersensitivity to antimycotic drugs that target cell wall or plasma membrane integrity in *C. glabrata *[10] and inviability in *S. pombe *[12]. In theory, the phenotypes of *kex2 *deletion mutants can be explained by the lack of processing events in substrate proteins rendering these dysfunctional, as in the case of the α-pheromone, where the lack of processing renders the *kex2 *mutant of *S. cerevisiae *mating deficient [3]. Because of the localization of the Kex2 protein in the late trans Golgi network [17] and an endocytic, prevacuolar compartment [18], it can be concluded that the target spectrum is limited to proteins attached to the cell surface, those proteins which are secreted into the environment or to the luminal domains of integral membrane proteins passing through these compartments. Accordingly, the phenotypes of *kex2 *mutants include the secretion of unprocessed protein precursors into the environment, e.g. the secretory xylanase of *T. reesei *[16]. However, these effects are blurred as the phenotypes observed from *kex2 *mutants may only be secondary effects themselves. Furthermore, missing Kex2-processing events may well be covered up by processing through other proteinases, such as the yapsins, a family of glycosylphosphatidylinositol (GPI) anchored aspartic proteinases [19,20]. In the case of proteinase pro-peptides these events may also occur autocatalytically, as proposed for CaSap2 [8]. While there is a fair number of proteins that have been annotated as potential Kex2 targets and two earlier studies have predicted Kex2 targets [9,10], the number of proteins for which experimental proof of cleavage by Kex2 exists, remains low.

Knowing the substrates of this proteinase would not only help to explain the phenotypes observed in fungal *kex2 *deletion mutants, but also provide insights into essential cellular regulatory mechanisms. We have aimed at providing an improved assembly of Kex2 target proteins and present first biochemical evidence for the processing of selected substrates, in particular from the human pathogenic yeasts *C. albicans *and *C. glabrata*. Furthermore, we provide evidence for extended subsite recognition in the P1'–P4' region. By using recombinant Kex2 proteinases and potential substrate proteins from pathogenic and non-pathogenic yeasts, we show that the substrate specificity is generally conserved between organisms. However, our data also suggest that some Kex2 proteinases have additional unique substrates.

## Results

### Heterologous expression and purification of Kex2 proteinases

**Figure 1:**
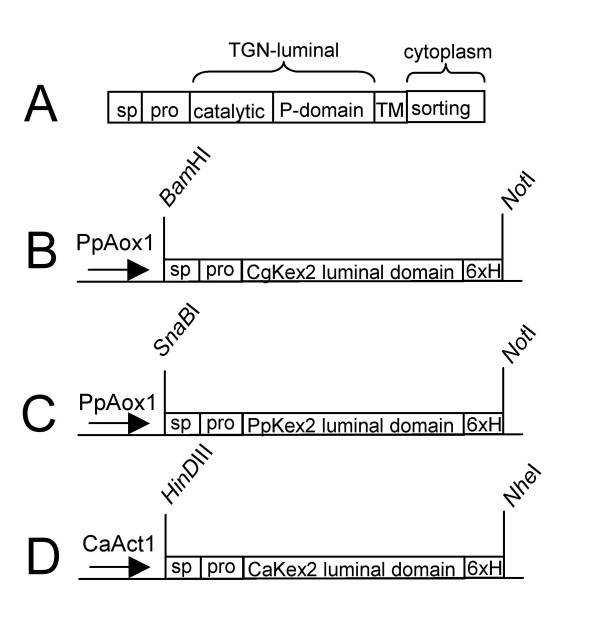
**Plasmid constructions for proteinase expression**. (A) Schematic representation of the domain structure of fungal Kex2 proteins. Kex2 consists of a signal peptide, an autocatalytically removed pro-peptide, a catalytic domain, a structural P-domain, a transmembrane domain and finally a cytosolic domain containing sorting signals to the Golgi apparatus. (B) For expression of *C. glabrata *Kex2 the part of the gene fused with a C-terminal 6 × His tag was cloned into pPic3.5 using the *Bam*HI and *Not*I restriction sites. (C) For expression of *P. pastoris *Kex2 the part of the gene fused with a C-terminal 6 × His tag was cloned into pPic3.5 using the *SnaB*I and *Not*I restriction sites. (D) For expression of *C. albicans *Kex2 the part of the gene fused with a C-terminal 6 × His tag was cloned into pCIp10 using the *Hin*DIII and *Nhe*I restriction sites.

In its native form Kex2 is a type I membrane protein with the catalytic domain located inside the trans Golgi network lumen (Figure 1A). As neither the transmembrane domain nor the cytosolic domain are necessary for the catalytic activity, it is possible to produce a soluble and secreted version of this enzyme by truncation of the gene just before the sequences encoding the transmembrane domain [21].

For the expression of the soluble forms of *S. cerevisiae*, *C. glabrata *and *P. pastoris *Kex2 enzymes the *P. pastoris *expression system (Invitrogen) was used. The strain expressing *S. cerevisiae *Kex2 was a kind gift of Guy Boileau [22]. For the expression of *C. glabrata *and *P. pastoris *Kex2 enzymes the 5' part of the gene coding for the luminal domain of the enzyme, including the native signal- and pro-peptide, plus a C-terminal 6 × His-tag were cloned into the pic3.5 vector (Figure 1B and 1C) and transformed into *P. pastoris *strain GS115. The transformants displaying the strongest extracellular proteolytic activity (ppCgKex2#12 and ppPpKex2#5) in test expressions were used for large-scale production of the enzymes.

Attempts to purify the *C. glabrata *and *P. pastoris *Kex2 enzymes via 6 × His-affinity chromatography were not successful, possibly due to burial of the epitope inside the protein. Thus, all three enzymes, including the one from *S. cerevisiae*, were purified to near homogeneity by a combination of anion exchange and size exclusion chromatography (Additional file 1).

Because several attempts to produce the intact, soluble form of Kex2 of *C. albicans *in the *Pichia *system failed, ultimately the native host *C. albicans *was used for production of this enzyme: the 5' part of the *C. albicans KEX2 *gene coding for the luminal domain of the enzyme, again including the native signal- and pro-peptide as well as a C-terminal 6 × His tag was put under the control of the constitutive and strong promoter of the *ACT1 *gene, as described under Methods (Figure 1D). The linearized plasmid was transformed into *C. albicans *strain CAI4 and the transformant giving the strongest Kex2-like activity in the supernatant (CaAct1-Kex2#7) was used for further large scale production of the enzyme, as above.

While we were able to produce the high Kex2 activity in supernatants, the efficiency of its purification remained low. Highest yields of enzyme were achieved using complex media including yeast extract and peptone, but this resulted in only impure enzyme preparations. However, the parental strain did not produce this activity (Additional file 1, Figure 1B). To avoid low-weight impurities in the enzyme preparations, which would have disguised product bands in further analytical experiments, the medium was passed over a 10 kDa size-exclusion column prior to the expression. In combination with the purification methods as outlined above this resulted in an enzyme preparation that contained only few other proteins and was devoid of low molecular weight contaminants.

### Activity testing of the enzyme preparations

Prior to use, enzyme preparations were adjusted to a common activity of one nmol/min per μl proteinase added in a standard reaction setup with the chromogenic substrate Z-Tyr-Lys-Arg-pNA. Neither preparations from a *P. pastoris *negative control strain nor from the *C. albicans *parental CAI4 strain displayed this activity (data not shown). In addition we performed controls with the *C. albicans *enzyme preparation to ascertain that the proteolytic activity was Kex2-dependent: The activity was indeed inhibited by PMSF, EDTA and ZnCl_2_, but not by pepstatin A (Additional file 1, Figure 1C).

To test whether the enzymes had similar properties we first tested the enzymes for optimal pH and temperature with the chromogenic substrate Z-Tyr-Lys-Arg-pNA. The optimal pH for all enzymes was between 7.2 and 7.4 (data not shown), as described earlier for the *S. cerevisiae *enzyme [23] and this pH was therefore used throughout all further experiments. In contrast, the result for the optimal temperature was surprising: all enzymes showed an elevated activity at unphysiological temperatures from 40°C to 50°C (data not shown), at which none of the source organisms display optimal growth, if any. Nevertheless, all following experiments were carried out at 37°C, reflecting human body temperature, as our main focus lay on the enzymes of the human pathogenic fungi *C. albicans *and *C. glabrata*.

**Figure 2:**
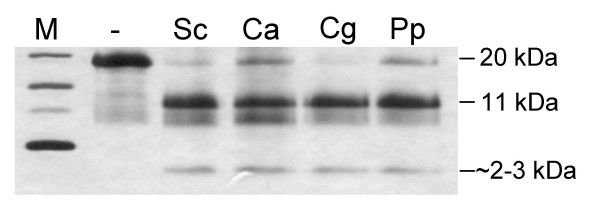
**Activity testing of the purified Kex2 enzymes**. The proteinases were tested with a proteinacious model substrate, the α-pheromone of *S. cerevisiae*. Digestion of the substrate protein (20 KDa) with the different proteinases resulted in the same expected pattern of products (11 and 2–3 KDa). Sc: *S. cerevisiae *Kex2, Ca: *C. albicans *Kex2, Cg: *C. glabrata *Kex2 and Pp: *P. pastoris *Kex2.

Since the *KEX2 *gene of *C. albicans *can complement the *kex2 *deletion in *S. cerevisiae *[8] and the *KEX2 *gene from *S. cerevisiae *can complement the loss of the *KEX2*-ortholog *KRP1 *in *Schizosaccharomyces pombe *[12], it is feasible to assume that these enzymes have similar to identical biological functions and biochemical properties. To show that this is also the case for the Kex2 proteinases from *C. glabrata *and *P. pastoris*, we tested whether all four proteinases cleaved the *S. cerevisiae *α-mating pheromone, a natural and proven substrate of Kex2 from *S. cerevisiae*, in a similar manner (Figure 2): The α-mating pheromone precursor protein was purified using the pET100-D *E. coli *expression system as described below for the other substrate proteins. Indeed, the four proteinases showed the same digestion pattern of the pheromone precursor into the expected fragments of the N-terminal 11 kDa peptide and peptides of 2–3 kDa size (Figure 2).

### Prediction of potential Kex2 substrates

Next, we developed a prediction method for potential Kex2 cleavage sites in substrate proteins to identify proteins from *C. albicans*, *C. glabrata *or *S. cerevisiae *for testing with the proteinases. Earlier studies [9,10] used very stringent search parameters and only looked in the N-terminal region of protein sequences. However, there is biochemical and biological evidence for processing of sites containing other amino acids in the P2 position [4,24] as well as activity on C-terminal motifs in other organisms such as the chloroperoxidase CPO of *A. niger *[25] and on membrane proteins such as Kex2 itself. Therefore we included Golgi-luminal portions of transmembrane proteins as well as full-length sequences of soluble proteins into our search. ER-retained proteins were excluded, as they should not come into contact with Kex2.

The predicted set of proteins was screened for potential cleavage sites using a position specific scoring matrix (PSSM) (Table 1, columns P4 to P1). This also allowed for a ranking of the sites found. The matrix used for the prediction of Kex2 substrate proteins was derived from systematic biochemical and genetic data generated with the *S. cerevisiae *enzyme [4,26,27]. All proteins with potential Kex2 cleavage sites were aligned with orthologous proteins of other fungi. This allowed for investigation of conservation of the potential cleavage site between different proteins with similar biochemical properties. This search yielded a total of 467 cleavage sites in 297 individual proteins (112 from *C. albicans*, 90 from *C. glabrata *and 95 from *S. cerevisiae*) which presumably pass the Golgi compartment. Selected substrate groups with conserved sites are shown in Additional file 2.

**Table 1:** A position specific scoring matrix for identification of potential Kex2 substrate proteins.

	P4	P3	P2	P1
A	0.25	0.64	0	0
C	0.05	0.71	0	0
D	0.05	0.27	0	0
E	0.05	0.62	0	0
F	0.60	0.99	0	0
G	0.05	0.70	0	0
H	0.73	1.00	0	0
I	0.28	0.55	0	0
K	0.72	0.60	0.50	0
L	0.09	0.90	0	0
M	0.53	0.71	0	0
N	0.49	0.69	0	0
P	0.20	0.01	0	0
Q	0.41	0.71	0	0
R	1.00	0.60	0.50	1.0
S	0.17	0.64	0	0
T	0.17	0.77	0	0
V	0.56	0.91	0	0
W	0.32	0.88	0	0
Y	0.47	0.85	0	0

The matrix represents the affinity of Kex2 towards the respective amino acid residues of positions P4-P1 relative to the cleavage site. Scores were calculated from biochemical and mating efficiency data given in the literature, as outlined in the text.

### Expression of substrate proteins

**Figure 3:**
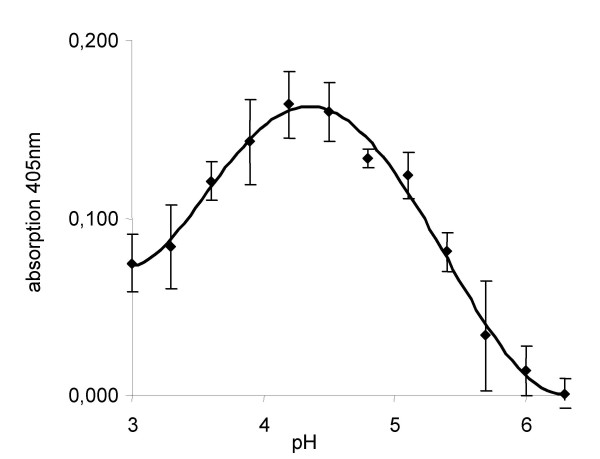
**Activity testing of refolded CaPho11**. CaPho11 was isolated from denatured inclusion bodies and refolded. To ensure its correct folding, activity testing using pNPP as a substrate was performed at different pH values. The enzyme was active and exhibited a maximum activity at pH 4.2–4.3.

From the 297 predicted potential Kex2 substrate proteins we selected a total of 43 proteins (three of *S. cerevisiae*, 26 of *C. albicans *and 14 of *C. glabrata*) for heterologous expression in *E. coli *(Additional file 3). These were chosen to cover a wide range of different cleavage sites and protein types and expressed using the TOPO-pET D100 system. The DNA fragments cloned were devoid of domains encoding signal peptides and putative GPI-anchor sequences. Out of these selected proteins, we were able to express and purify thirteen from *C. albicans*, ten from *C. glabrata *and one from *S. cerevisiae *(the α-pheromone mentioned above). Since the majority of the chosen proteins accumulated as inclusion bodies, we converted these proteins into a soluble form by on-column refolding. To test for overall correct folding of the refolded proteins, we performed an activity test for the substrate CA5147, an acid phosphatase, which was the only protein with a known activity in this set. Indeed, we were able to confirm the activity of this protein and observe a maximum activity at pH 4.2–4.3 (Figure 3) using para-nitrophenol phosphate as a substrate. This shows that at least some refolded protein assumes its native structure and can thus be used for specific proteolysis assays.

### *In vitro *proteolytic processing of substrate proteins by Kex2 from *C. albicans*, *C. glabrata*, *S. cerevisiae *and *P. pastoris*

**Figure 4:**
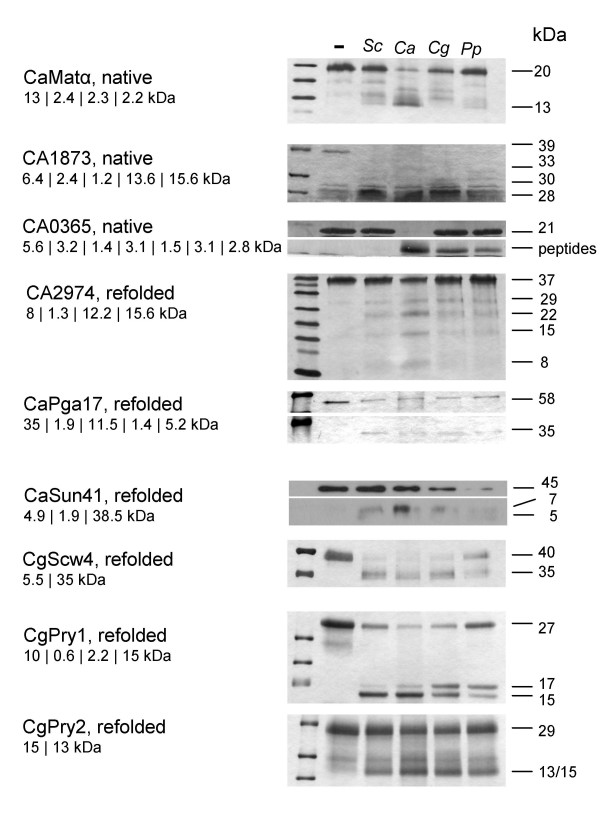
**Proteolytic digests of putative substrate proteins**. (A) Recombinant substrate proteins were digested with each of the four proteinases. -: neg. control, Sc: *S. cerevisiae *Kex2, Ca: *C. albicans *Kex2, Cg: *C. glabrata *Kex2 and Pp: *P. pastoris *Kex2. Potential fragment sizes are given in kDa underneath the names (vertical bars: potential cleavage sites). All digests were visualized in silver stained gels, except CaSun41, where the N-terminal X-press epitope was detected in a Western blot. Proteins are digested at all major substrate sites found in the sequence and for most, intermediate products can be observed. Proteins not hydrolysed by Kex2 are not depicted (see text). Substrate CA0365 is not processed by ScKex2, but by all other proteinases, most efficiently by CaKex2.

Very few studies provided experimental evidence that predicted Kex2 cleavage sites in potential substrate proteins are in fact processed by Kex2 proteinases. In order to determine susceptibility of the purified proteins to proteolytic processing by Kex2, all potential substrate proteins purified above were digested with each of the four proteinases. A selection of digestions is depicted in Figure 4. Based on the scores given by the algorithm, we expected most proteins to be cleaved. Indeed, we observed rapid cleavage at the predicted cleavage sites for 2/3 of the proteins. This also included cleavage at sites with lower scores in polypeptide precursors (e.g. CaEce1, position 92, Additional file 2). In contrast, some proteins were not cleaved even though they contained sites with high scores, such as CaCcw14 (see Additional file 2). Furthermore, one protein (CA0365) was cleaved very differently by the proteinases: while it was not cleaved at all by ScKex2, CaKex2 rapidly processed the precursor into peptide sized fragments, without any noteworthy appearance of intermediates under the standard reaction conditions (Figure 4). A similar activity was observed with CgKex2 and PpKex2, while at a considerably slower rate of hydrolysis.

Proteins that were cleaved into fragments of the expected sizes were CA0365, CaEce1 (CA1402), CA1873, CA2974, CaPga17(CA4679), CaTos1 (CA2303), CaSun41 (CA0883), CgScw4 (CAGL0M13805g), CgSUN4 (CAGL0L05434g), CgPir1 (CAGL0M08492g), CgPry1 (CAGL0F05137g) and CgPry2 (CAGL0G07667g). Proteins that remained fully uncleaved were CaCcw14 (CA2942), CaPho11 (CA5147), CaRbt4 (CA0104), CaCrh1 (CA0375), the Plb-homolog CAGL0J11770g and the three proteins of unknown function CA1394, CAGL0H08910g and CAGL0A02277g.

**Figure 5:**
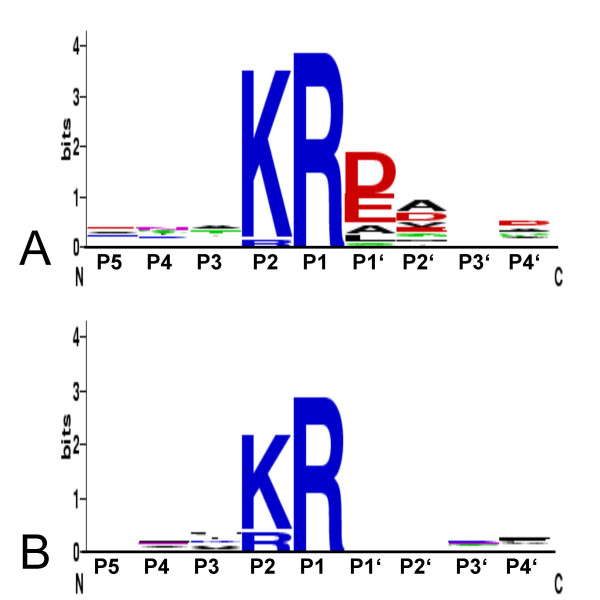
**Statistical sequence analysis of predicted Kex2 cleavage sites**. (A) sequence logo of cleaved sites and (B) of non-cleaved sites. Position 5 of the logos corresponds to the P1 position in the substrate. Negatively charged residues (red) are overrepresented in processed substrate P1', P2' and P4' positions. Color key: red: negatively charged, blue: positively charged, black: apolar, green: polar.

The pattern of cleavage vs. non-cleavage observed was not sufficiently explained by the score calculated from the prediction algorithm among the proteins tested. Therefore, we inspected the amino acid distribution surrounding the investigated and other known cleavage sites for other patterns: indeed, a high overrepresentation of negatively charged (aspartic/glutamic acid) and small (alanine, valine, leucine) residues in the P1', P2' positions and a similar moderate overrepresentation in the P4' position was found, while positively charged residues were underrepresented at those substrate sites which were digested. In the case for the sites not cleaved, no over- or under-representation was observed (Figure 5).

### Reflection of substrate recognition in proteinase structure

Next, we asked whether the apparent preference for negatively charged residues in the P1'–P4' region of substrates digested by Kex2 proteins is reflected by the structure of the proteinases in the substrate binding cleft.

**Figure 6:**
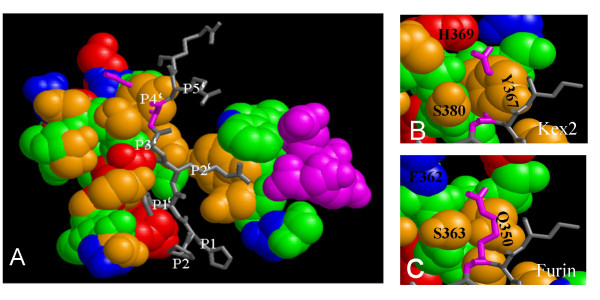
**Investigation of the three dimensional models of Kex2, furin and Kumamolisin for substrate binding properties**. (A) Superimposed 3D coordinate sets for the three proteases reveal colocalization of the Kumamolisin propeptide residues with the predicted S1' and S2' binding pockets in furin and Kex2. A region identified for binding of the inhibitor Eglin-c (purple) [31] is not involved in binding of the propeptide. (B) and (C) A potential S4' binding pocket is identified which is terminated by H369 in Kex2 and E262 in furin. Numbering "a" through "E" refers to residues used in Table 2, which lists the respective binding pockets and references.

Recently, a 3D model of the bacterial subtilisin kumamolisin of *Bacillus *novospec was published [28]. The enzyme studied there was incapable of autoproteolytic activation thus retaining the pro-peptide. By superimposition with the coordinate sets of *S. cerevisiae *Kex2 [29] and *Mus musculus *furin [30] we were able to investigate the potential substrate binding pockets in the P1' – P4' region (Figure 6A) as outlined by the intact pro-peptide cleavage site still bound into the substrate binding cleft. Indeed, the P4-P1 positions of the Kumamolisin pro-domain aligned with the known S4-S1 pockets of the enzymes (not shown), as well as the P1'–P3' positions with the S1'–S3' pockets predicted in the literature [31,32] (Figure 6A).

The neighbouring S1' and S3' pockets are characterized by positive charges in ScKex2 (H213, H381) as well as in furin (R193, H194, H364), and both pockets may well accommodate aspartate or glutamate residues in the substrate. In furin, the excess charge possibly results in a stronger selection for negatively charged residues in the P1' position, but as the S2 pocket is directly adjacent to the S1' pocket, the lack of a positively charged P2 residue in furin substrates may compensate this effect. The S2' pocket, located on the opposite side of the cleft, as well contains a terminal positive charge (R318 in ScKex2, R298 in furin) which would favour negatively charged residues in the P2' position.

A potential P4' pocket was also identified (Figure 6B and 6C). The P4' residue aligns between S363 and Q350 and extends towards E362 in the furin model (Figure 6C). The alignment with ScKex2 is of lesser quality in this region, but nevertheless a similarly built potential binding pocket is seen in the ScKex2 enzyme bordered by S380 and Y367 (Figure 6B). However, the equivalent to the negative terminal charge of E362 in furin would be the positive charge of H369 in ScKex2.

In summary, the structure of the enzymes explains the increased preference for negatively charged P1'–P4' residues in the substrates.

### Conservation of residues involved in substrate recognition

**Figure 7:**
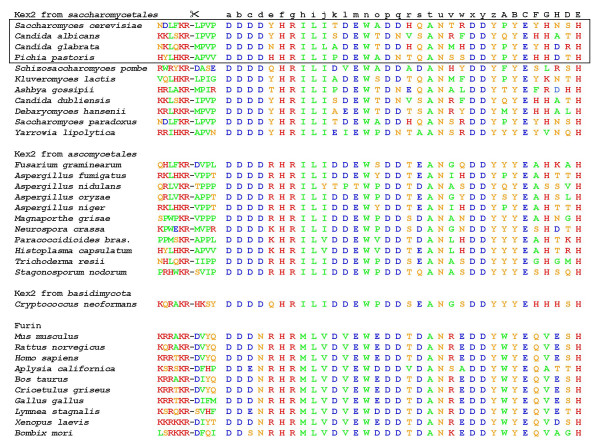
**Sequence Alignment of fungal Kex2-like proteins**. A protein sequence alignment of the residues involved in substrate specificity determination shows that the electrostatic properties of the binding regions are highly conserved. Red: positive charges, blue: negative charges, orange: polar residues, green: apolar residues, : Propeptide cleavage site, numbering "a" through "E" refers to residues used in Table 2, which lists the respective binding pockets and references.

It is known from previous studies, that *C. albicans KEX2 *can complement *KEX2 *in *S. cerevisiae *[8] and this gene in turn can complement the *KEX2 *ortholog *KRP1 *in *S. pombe *[12]. Therefore, it must be concluded that the corresponding proteinases have similar substrate specificities and activities. Nevertheless, we have been able to show that at least in the case of one substrate (CA0365) the proteinases of *S. cerevisiae *and *C. albicans *behave differently. To investigate whether this difference as well as the question whether or not the substrate specificity in general is the same in different fungi, we generated a sequence alignment of Kex2-orthologous proteins from fungi and furin-orthologous proteins from mammals (Figure 7) and investigated the residues involved in substrate recognition (Table 2) for their degree of conservation between the different species.

**Table 2:** Compilation of residues relevant for substrate recognition in *S. cerevisiae *and *M. musculus *furin.

#	ScKex2	MmFurin	Pocket	Reference
a	D175	D153	S2	[29]
b	D176	D154	S2	[29]
c	D210	D191	S2	[29]
d	D211	N192	S2	[29]
e	Y212	R193	S3' S1'	[31,32] This work
f	H213	H194	S1'	[32]
g	R216	R197	S3'	[32]
h	I245	M226	S4	[29]
i	L246	L227	S4	[29]
J	I250	V231	S4	[29]
k	T252	D233	S4, S6	[67]
l	D254	V235	S4	[29]
m	E255	E236	S4	[29,67]
n	W273	W254	S4	[29]
o	A276	E257	S3, S5	[32,29]
p	D277	D258	S1	[29]
q	D278	D259	S5	[32]
r	H281	T262	S5	[32]
s	Q283	D264	S4, S6	[67]
t	A311	A292	S1	[29]
u	N314	N295	S1	[68]
v	T317	R298	S2'	[31]
w	R318	E299	S2'	[31]
x	D320	D301	S1	[29]
y	D325	D306	S1	[29]
z	Y327	Y308	S4	[31]
A	P347	W328	S2'	[31,32]
B	Y348	Y329	S2'	[31]
C	E350	E331	S1	[29]
F	Y367	Q350	S4'	This work
G	H369	-	S4'	This work
H	N379	E362	S4'	This work
D	S380	S363	S3' S4'	[31] This work
E	H381	H364	S1'	[32]

Residues relevant for substrate recognition given in the literature or identified in this study were compiled. Column a: Residues were numbered "a" trough "E". The identical numbering was also used in Figure 6 and Figure 7; columns 2 and 3: The respective analogous residues in ScKex2 (Genebank accession no. CAA96143) and MmFurin (Genebank accession no. CAA37988) are given along with the reference.

The S1 pocket (composed of positions p, t, u, x, y and C) is fully conserved and among fungi this is also true for the four negative charges of the S2 pocket (positions a, b, c and d). Interestingly, we observed for the S4 and the S1' position that the enzymes from *Ascomycetales *combine the charge-selective properties of the *S. cerevisiae *Kex2 enzyme with those from the furin enzymes, and thus probably display the most discrete substrate recognition. Among the *Saccharomycetales *the residues are conserved for the major subsites S4, S2, S1 and S1' with minor exceptions only. Differences are visible in subsites where there is no strong selection to or discrimination against substrate residues, such as the S5 pocket (positions q and r). The S2' pocket is generally positively charged, however, this charge is mediated by one histidine in either the v or the w position.

In summary, it is seen, that the substrate selectivity among *Saccharomycetales *Kex2 enzymes is very conserved, and that there are no substitutions that would explain the differential processing of substrate CA0365 between the four proteinases. Therefore, the enzymes must discriminate their substrates either through further subsites or through processes independent of the primary sequence surrounding the cleavage site.

### Relevance of substrate structural features for cleavage

**Figure 8:**
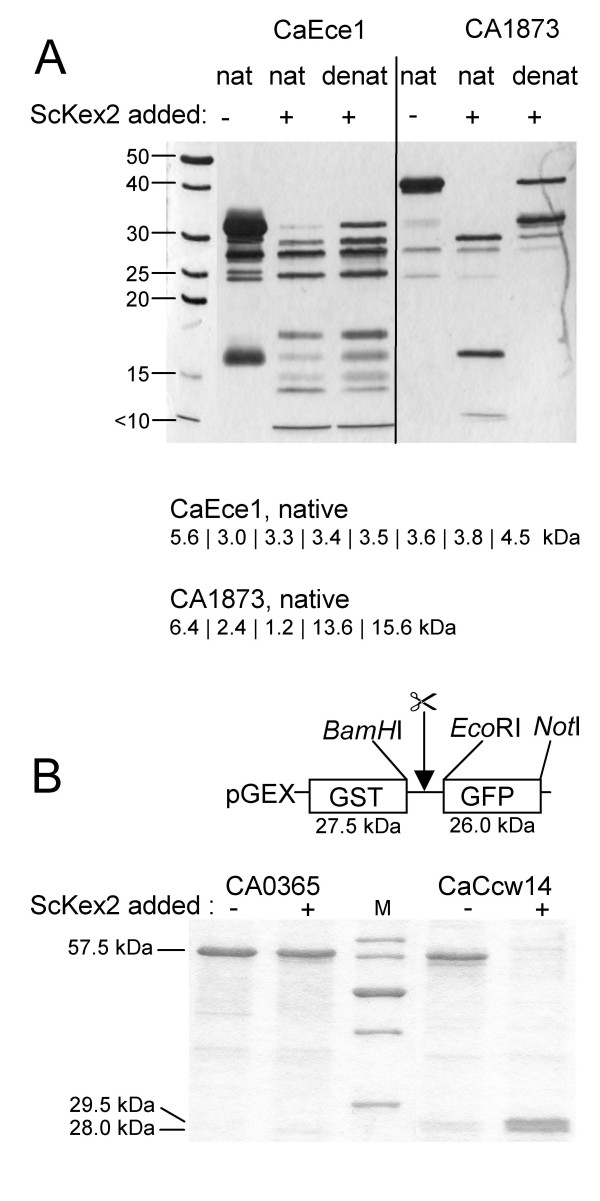
**Relevance of proper folding for proteolysis**. (A) Sites readily cleaved in the native protein (nat) are cleaved less in heat denatured protein (denat). Shown are the results for CaEce1 and CA1873. (B) Potential sites not cleaved in the native protein are cleaved when exposed to the environment by fusion between GST and GFP (see text).

During the *in vitro *cleavage experiments, we observed that proteins purified from the soluble fraction of *E. coli *lysates were generally processed more efficiently than those purified and refolded from inclusion bodies. Therefore, we predicted that the three dimensional structure of the substrate and the exposure of the putative processing site on the protein surface is crucial for processing to occur. To investigate this further, we tested if sites that were readily cleaved in the native protein were still cleaved in a denatured form of the protein: two substrate proteins that were readily cleaved by ScKex2 (CaEce1 and CA1873) were heat denatured prior to addition of the ScKex2 proteinase (Figure 8A). As expected, both were cleaved less in the denatured form. This effect is more pronounced for CA1873 than for CaEce1, as CaEce1 contains seven equal cleavage sites and is thus generally more prone to processing than CA1873. The reduced cleavage of partially denatured/refolded proteins can be explained by either inaccessibility of the site due to burial in the denatured structure or by the failure to form a specific secondary structure needed for processing.

Also, we did not observe cleavage for all proteins with potentially good sites. Therefore, we tested if this was due to an uncleavable primary sequence or if there were structural constraints preventing cleavage: site 3 of CaCcw14 and site 1 of CA0365, were each fused between a GST and a GFP domain and so exposed to the solvent. The GST-CA0365 [1]-GFP fusion protein was not cleaved (Figure 8B, lane 2), indicating that this sequence is not a substrate of ScKex2 and the non-cleavage of the full length protein is not due to structural constraints, as was expected due to the cleavage by the other three Kex2 enzymes. In contrast, the GST-CaCcw14 [3]-GFP fusion protein was readily cleaved by ScKex2 (Figure 8B, lane 5), demonstrating that this primary sequence reflects a good substrate and the non-cleavage in the full length protein must be due to structural constraints. This gives further evidence that accessibility and/or secondary structure of the cleavage site are essential for processing.

## Discussion

The pleiotropic phenotype of fungal Kex2 deletion mutants is attributed to the lack of posttranslational, proteolytic activation of substrate proteins. Besides biochemical data describing the P4-P1 substrate recognition towards short peptides of the *Saccharomyces cerevisiae *enzyme, only very few data exist of substrate preferences of fungal Kex2 proteins. Several proteins have been discussed as "potential Kex2 substrates", however there is no experimental data confirming actual cleavage by Kex2, except for a few cases, e.g. killer toxin, α-mating pheromones and proteinase propeptides. In the present study, we have investigated cleavage of recombinant Kex2 proteinases on recombinant, potential Kex2 substrates in order to get a first insight into the possible substrate repertoire of these regulatory proteases.

For heterologous production of soluble Kex2 enzymes, we selected the proteins from the two pathogenic fungi *C. albicans *and *C. glabrata*, as the phenotypes of the respective deletion mutants include avirulence [9] and increased susceptibility to antifungal compounds [10]. In addition, we selected the well characterized *S. cerevisiae *enzyme and the ortholog from *Pichia pastoris*, as this enzyme is often involved in the heterologous production of secretory proteins. The Golgi-luminal domains of these four enzymes were expressed in the host *P. pastoris *and purified from culture supernatant, except for Kex2 from *C. albicans*, which was produced in *C. albicans *itself, as it was not expressible in *Pichia*. The purified enzymes showed similar pH- and temperature dependencies: the optimal pH was found at pH 7.2, as reported for *S. cerevisiae *Kex2 [23], but surprisingly maximum cleavage of the artificial substrate Z-TKR-pNA was observed at unphysiological temperatures ranging from 40°C to 55°C. The fact that the enzymes retain their catalytic activities at theses temperatures could reflect a stabilizing effect on the protein structure proposed for the P-domain of Kex2 [33].

To identify new substrates of Kex2, we have searched the genomes of *C. albicans, C. glabrata *and *S. cerevisiae*, for secretory proteins containing potential cleavage sites. These were grouped into clusters by sequence similarity and based on the conservation of such sites selected for heterologous expression and *in vitro *cleavage testing by Kex2 enzymes (Additional file 2).

All four proteinases cleaved the *S. cerevisiae *α-mating pheromone precursor in the same expected pattern, confirming the orthologous enzymatic activities of the proteins. As it is known, that the *C. albicans *and *S. pombe *Kex2 proteins can complement the *S. cerevisiae *Kex2 protein *in vivo *(30, 38), it was not surprising that almost all substrates were cleaved (or *not *cleaved) in an identical manner. However, one substrate (CA0365, Figure 4) was differentially processed. This demonstrates that even though the proteins have very high sequence similarity they still have partially different substrate preferences.

Statistical sequence analysis of processed vs. non-processed sites reveals an overrepresentation of negatively charged (aspartic/glutamic acid) or small residues in the P1', P2' and P4' positions, which has also been reported for substrates of the mammalian furin/PC proteinase family [34] (Figure 5). This finding is strengthened by the fact that a mutant of ScPir4, where the Kex2 cleavage site was changed from KR/D to KR/A failed to undergo processing [35]. Previous biochemical analyses of substrate preference have focussed on the S1–S4 regions of the enzymes [4-6], due to the nature of the substrates used in those studies. However, the solved three dimensional structures of *S. cerevisiae *and *Mus musculus *furin in complex with proteinaceous inhibitors such as Eglin-c have lead to the postulation of binding pockets also in the S1' and S2' regions [31]. In order to identify further residues involved in substrate recognition in the S1'–S4' region, we have produced a structural alignment of *S. cerevisiae *Kex2, *M. musculus *furin and the bacterial Subtilisin-like proteinase kumamolisin of *Bacillus *novospec MN-32 [28]. The latter structure was solved for an active-site mutated form of the protein, which still retained its propeptide. Due to the autocatalytic nature of the maturation process of subtilisin-like proteinases [28], the propeptide is the first substrate cleaved by the enzyme and should reflect an optimal substrate. Indeed, the P1' residue of the Kumamolisin propeptide aligned with the predicted S1' binding pocket of the kexins (Figure 6). In addition, we identified a potential S4 binding pocket, which in Kex2 terminates with the positively charged H369 (Figure 6B).

A sequence alignment of residues involved in substrate recognition shows that these residues are generally very highly conserved among the enzymes investigated here (Figure 7). Accordingly, there is no single residue that could explain the strong difference between ScKex2 and CaKex2 in cleavage of substrate CA0365. However, it is possible that a combination of such amino acid exchanges could generate such an effect. In accordance with the experimental data, it is likely that the Kex2-ortholog enzymes of the *Saccharomycetales *exhibit a similar activity and the cleaved substrate pattern is comparable within these. However, for the enzymes from *Ascomycetales *it would be expected that they are more stringently selective for charged residues in the P4 and P1' position.

In addition to the very important direct enzyme-substrate interactions outlined here, other parameters must influence substrate recognition by Kex2 proteinases: the reduced cleavage of heat denatured protein shows that a site must be properly folded to be accessible. This view is strongly supported by the fact, that a potentially preferred substrate (CaCcw14) remains uncleaved in its native context but becomes cleavable, when exposed to the proteinase in a fusion protein (Figure 8). In our experiments 1/3 of the selected proteins remained uncleaved. Hence, to properly identify proteinase substrates, it is essential to include further parameters such as substrate structure in addition to primary sequence into the prediction algorithm.

Our data provide information beyond those previous data based on *in silico *predictions or assays with small peptides only. By using heterologous expressed proteases and substrates we were able to show the potential of each of the investigated Kex2 enzymes to digest selected putative substrates. However, further *in vivo *experiments are necessary in future studies to undoubtedly infer proteolytic maturation of these substrates.

Aside from α-mating pheromone- and killer toxin precursors, the only previously experimentally proven Kex2 substrates are the glycolytic enzymes Exg1 of *S. cerevisiae *[36] and Xylanases of *T. reesei *[16], the aspartic proteinase CaSap2 [8], the structural cell wall Pir protein family [37] and the hydrophobin Rep1 of *Ustilago maydis *[38]. In our experiments we were able to confirm processing by Kex2 for the cell wall modulating enzymes CaSun41 (CA0883), CgScw4 (CAGL0M13805g) and CgSun4 (CAGL0L05434g) and for CgPir1, which had been predicted to be Kex2 substrates in earlier *in silico *searches [9,10]. Additionally, we observed *in vitro *cleavage for several proteins which have not previously been discussed as Kex2 substrates such as CaEce1, a group of Ops4-like proteins and two members of the Pry-protein family.

In our tests three proteins of the "plant pathogenicity related" Pry-protein family (CaRbt4, CgPry1 and CgPry2) were included. The proteins of this family contain a strongly conserved KR-motif (see Additional file 2), but the proteins are not cleaved in a similar pattern: While CgPry1 is cleaved efficiently, CaRbt4 is not cleaved at all and CgPry2 only very slowly. It is therefore likely, that the conserved site of the Pry proteins is not cleaved in the fully native protein, and that processing of CgPry1 only takes place in the additional sites not present in the other two proteins.

The major phenotype described for *kex2 *deletion mutants in *Candida *revolves around morphological defects of the cell wall [8] and the resulting hypersensitivity to compounds interfering with the surface integrity [10]. Several Kex2 target proteins directly interact with the fungal cell wall or are structural components thereof: the Pir proteins, glucanases such as Exg1, or proteins of the Sun/Scw family. While the direct consequence of failure to mature is not known for these proteins, the phenotypes of the respective deletion strains resemble those of *kex2 *deletion strains: mutants lacking cell wall localized glucanases such as ScExg1 [36] or CaBgl2 [39] and mutants lacking members of the Pir [40] or the SUN-family [41,42] show similar increased sensitivities towards several cell wall or membrane perturbing compounds [8,10]. Here it is interesting, that the Kex2 cleavage site is found in several but not in all glucanases.

Additionally, Pir deletions result in the formation of cell aggregates [40], which is also be seen in the *S. cerevisiae sun4 *and *C. albicans sun41 *deletion strains [41-43] and are also observed in *C. glabrata kex2 *deletion strains (data not shown). Furthermore, a *S. cerevisiae scw4/scw10 *double mutant [44] and a *C. albicans sun41 *strain showed enlarged cells [42], a phenotype which can also be observed in the *C. glabrata kex2 *mutant (data not shown). Furthermore, calcofluor white stained *C. albicans kex2 *cells show an abberant staining pattern [8], which would be in agreement with the potential changes in chitin deposition as seen from the abberant septum processing in *C. albicans sun41 *strains [41]. The Kex2 cleavage site in Sun4- and Scw10-like proteins is preceded by an N-terminal stretch of positively charged amino acids, mainly histidines (see Additional file 2). This feature, which we termed "His-Box", is also found in Tos1 proteins, only here it is located further inside the protein and is additionally preceded by another Kex2 cleavage site. It can be speculated that, if this motif was involved in cell wall attachment, processing would lead to differential localization of the mature protein, e.g. secretion as observed in *C. albicans *for Sun41 and Tos1 [42].

**Figure 9:**
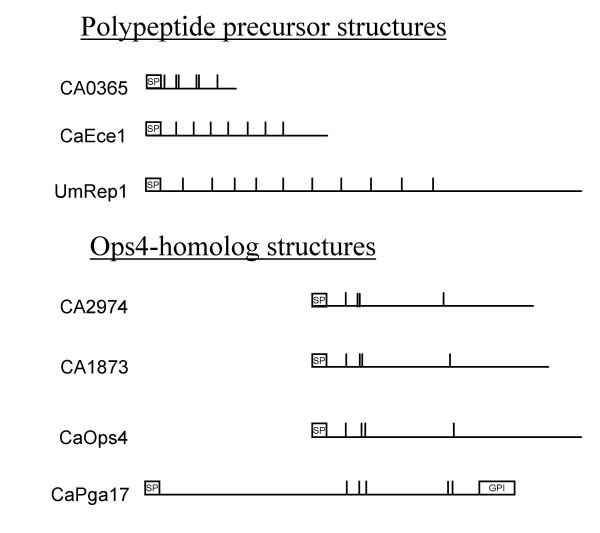
**Schematic representation of polypeptide- and Ops4-like substrates**. Kex2 cleavage sites are represented by vertical bars. SP: Signal peptide, GPI: potential GPI anchor attachment site. The proteins are digested at all sites found (see Figure 4 and Figure 8).

Besides explaining previously observed phenotypes, the identification of cleavage sites may yield additional functional information about a protein: the expression of CaEce1 is tightly associated with hyphae in *C. albicans*, but the deletion has no apparent effect on morphology and no function could be assigned to this protein [45]. While there is no sequence homology, the polypeptide precursor structure of CaEce1, and also that of CA0365, resemble that of the repellent protein Rep1 of *Ustilago maydis *[38]. The UmRep1 protein contains ten strongly conserved repeats separated by Kex2 cleavage sites and a longer terminal fragment with no similarity to the repeats (Figure 9). CA0365 is shorter, with only three conserved repeats each containing another internal Kex2 cleavage site, but no terminal fragment. In CaEce1, the seven repeats are less conserved, but the longer, terminal fragment is present. UmRep1 functions as a structural component of aerial hyphae and CaEce1 or CA0365 might play similar roles on the hyphae of *C. albicans*. All three proteins seem to have in common that a processing via Kex2 proteinases may be necessary for their proper biological function.

A second group of proteins without assigned function identified as Kex2 substrates is the family of *C. albicans *Ops4-like proteins, whose members are differentially regulated in white-opaque switching [46] and mating [47]. This family consists of CaOps4, CA2974, CA6162, CA1873 and CaPga17 (Figure 9). The *C. albicans *and *S. cerevisiae kex2 *deletion mutants are mating deficient [3,48]. This has been attributed to the lack of processed α-mating pheromone, but if the above proteins are indeed involved in the mating process, the *kex2 *mating deficiency could be more severe than thought.

## Conclusion

In summary, our data show that fungal Kex2 proteinases are similar in their substrate activities but these substrates may have different functions according to the different biological backgrounds of the investigated fungi, including pathogenicity in humans. In addition, the preferred processing sites of these substrates do not only depend on the amino acids surrounding the processing site, but also on other features such as three dimensional structure. Furthermore, Kex2 proteinases may have unique substrates whose processing sites are adapted to individual proteinases in each organism.

## Methods

### Oligonucleotides

Oligonucleotides (TIBMolBiol, Germany) used for cloning of expression vectors in this study are given in Additional file 4.

### Heterologous proteinase expression in *Pichia pastoris*

*Candida glabrata *and *Pichia pastoris *Kex2 enzymes were expressed using the *Pichia *expression system (Invitrogen) according to manufacturer's instructions. Briefly, the DNA coding for the Golgi-luminal part of the protein was PCR-amplified from genomic DNA with oligonucleotides containing terminal restriction sites (*Bam*HI/*Not*I and *Sna*BI/*Not*I, respectively) and a sequence for a C-terminal 6 × His-tag, cloned into the pic3.5 vector and transformed into *Pichia *strain GS115 using an optimized electroporation protocol [49]. Transformants were screened by testing of enzymatic activity against the chromogenic substrate Z-Tyr-Lys-Arg-pNA (see below) in the supernatant of pilot expressions and the clone exhibiting maximum activity used for scale-up. For large-scale production, cells were grown in 500 ml buffered minimal glycerol medium at 30°C over night, harvested by centrifugation, washed and resuspended in 50 ml buffered minimal methanol medium. Maximum activity was detected after 16 h of growth, after which the culture supernatants were harvested and the recombinant enzymes purified as described below.

### Heterologous proteinase expression in *Candida albicans*

The soluble form of *C. albicans *Kex2 was expressed in the native host, as several attempts of heterologous expression in *P. pastoris *failed. The *KEX2 *gene was PCR-amplified from genomic DNA of *C. albicans *with primers containing restriction sites (*Hin*DIII/*Nhe*I) and the sequence for a 6 × His-tag, cloned into pCIp10 [50] and thus put under control of the mainly constitutive *C. albicans ACT1 *promoter. The plasmid was linearized with *Nco*I and transformed into *C. albicans *strain CAI4 using the same protocol as above for *Pichia *transformation. Transformants were selected on minimal medium and screened using the supernatant of 5 ml YPD (1% yeast extract, 2% peptone, 1% glucose) over night cultures for testing of enzymatic activity as above. For preparative expression, a 500 ml YPD culture was grown over night, the cells harvested, washed twice with 50 ml YPD, resuspended in 50 ml YPD and further cultivated at 30°C. Maximum Kex2 activity in the supernatant was observed after 12 h of growth at 30°C, after which the supernatant was collected and the recombinant protein purified as described below. YPD medium used for expression was previously freed from low molecular weight impurities by passing over a 10 kDa size exclusion Centricon-20 column (Millipore),

### Purification of secreted soluble Kex2 proteins

To purify the recombinant enzymes, 50 ml sterile filtered expression culture supernatant were concentrated on a 30 kDa size-exclusion Centricon-20 column (Millipore) to a volume of approximately 1–2 ml, desalted using a PD-10 column (Amersham Biosciences) and eluent diluted to a volume of 20 ml into IAEX buffer (50 mM BisTris pH 4.5, 10 mM NaCl). This was loaded onto an HiTrap ANX FF anion exchange column (Amersham Biosciences), washed, and eluted with IAEX buffer containing 100 mM NaCl. The eluent was then again concentrated, the buffer changed into storage buffer (50 mM BisTris, pH 7.2 50% w/v glycerol) and the enzymes kept at -20°C.

### Proteinase activity quantification

Proteolytic activity of the purified enzymes was assayed using the chromogenic substrate Z-Tyr-Lys-Arg-pNA (Bachem, Switzerland) as described previously [23]. Assays were done in buffer containing 50 mM BisTris (pH 7.2), 1 mM CaCl_2_, 0.5 mM substrate in a total volume of 100 μl at 37°C. For the measurement of time kinetic data, the reaction was started by mixing 50 μl of solution containing the proteinase with 50 μl containing the substrate. The temperature gradient for optimal reaction temperature measurement was generated in a thermocycler (Biometra) and the reaction terminated by the addition of EDTA to a final concentration of 10 mM. Liberation of p-nitroannilide (pNA) was measured at 405 nm in a spectrophotometer (Tecan). All measurements were calibrated against negative controls without proteinase and repeated at least three times.

### *In silico *identification and analysis of substrate sequences

The protein sequence sets analysed here were downloaded from the Genolevures Website [51,52], from CandidaDB [53,54] and from the Stanford Genomic Resources FTP server [55]. Sequence analysis on genome-scale was done using custom perl scripts within the bioperl framework, incorporated into a local database, as described in the results section. Entry into the secretory pathway and membrane topology were predicted with the Phobius algorithm [56]. Sequence logos were created using the Weblogo website [57]. All programs were run under SUSE Linux 10.1.

### Heterologous expression, renaturation and purification of substrate proteins

Heterologous expression of substrate proteins was done using the pET100-D TOPO vector system (Invitrogen) in *E. coli*, strain Rosetta (Novagen). Either, exponentially growing cells were induced with IPTG and grown for 3 h in a volume of 50 ml or autoinduced by growth in 50 ml LB containing 0.05% glucose and 0.2% lactose [58]. Harvested cells were lysed in 5 ml BugBuster (Novagen) with Benzonase nuclease (Novagen) and lysosyme (Sigma) added according to the manufacturers' description. Proteins expressed in a soluble form were purified using 6 × His chelating chromatography and analyzed by SDS-PAGE. Proteins expressed in form of inclusion bodies were refolded using β-cyclodextrin [59]. Inclusion body pellets were dissolved in solubilization buffer (100 mM Na_2_HPO_4_, 100 mM NaCl and 8 M urea) at 60°C. The denatured protein was bound to Ni-Agarose and washed with 20 bed volumes of solubilization buffer. The urea was thoroughly removed with buffer A (100 mM Na_2_HPO_4_) containing 0.1% Triton X-100 (Sigma). Excess Triton X-100 was removed by washing with 10 bed volumes of buffer A and the bound protein refolded over night in buffer A containing 5 mM β-cyclodextrin (Sigma). The refolded, bound protein was then again washed with 20 bed volumes buffer A and eluted with 200 mM imidazole and 0.001% Triton X-100. The eluent was passed through a 20 μm sterile filter to remove aggregates and then analyzed via SDS-PAGE.

To confirm proper folding by this method we tested one of the proteins, a putative acid phosphatase, for activity. One μl each of the refolded protein solution was assayed in 100 mM NaAcetate buffered from pH 3 to 6 at room temperature towards its activity against 0.5 mM para-nitrophenol phosphate (pNPP). Liberation of p-nitrophenol was measured in a spectrophotometer (Tecan) at 405 nm and the experiment repeated three times.

### Proteolytic digestion of substrate proteins and detection of digestion products

Proteolytic digests of Kex2 substrates were performed in Kex2 buffer (20 mM Tris-HCl, 1 mM CaCl_2_, pH 7.2, modified from [60]) at 37°C. To observe intermediate products in the case of proteins with several potential cleavage sites, time series of the reactions were conducted by stopping the reaction of aliquots by fast heating to 95°C. Depending on the expected product sizes, the proteolytic digests were resolved either on Tris-glycine or Tris-tricine [61] polyacrylamide gels and visualized by silver staining and/or western blotting using an antibody against the N-terminal Xpress epitope (Invitrogen).

### Alignment of proteinase structures and sequences

Sequences were aligned locally with the ClustalW algorithm [62] and edited with the BioEdit software [63]. Coordinate sets for proteinase structures were downloaded from the Protein Data Bank [64], superimposed using the MASS algorithm [65] and visualized with RasMol [66].

## Authors' contributions

OB carried out the experimental and *in silico *work and wrote the manuscript. YK expressed the *P. pastoris *Kex2 enzyme. BH conceived the study and was involved in writing the manuscript. All authors read and approved the final manuscript.

## Supplementary Material

Additional file 1 **Purity of Kex2 preparations**. (A) Preparations of *C. glabrata *(Cg), *S. cerevisiae *(Sc) and *Pichia pastoris *(Pp) soluble Kex2 enzymes were pure, as shown by silver staining. (B) To ensure, that the observed proteolytic activity observed in culture supernatant of the CaKex2 expressing strains was not due to another secreted protease, the activity was monitored in parallel in the parental wild type (SC5314). The wild type did not have this activity. (C) Furthermore, the activity against the substrate CA0365 from the CaKex2 expressing strain was inhibited by PMSF, EDTA and ZnCl2, but not by Pepstatin A, an inhibitor of the secretory aspartic proteinases of *C. albicans*. Also shown (first lane) is the missing activity of ScKex2 against this substrate. Click here for file

Additional file 2 **Selected groups of predicted substrates with conserved cleavage sites**. Selected groups of proteins with conserved predicted Kex2 cleavage sites are listed. Potential Kex2 cleavage sites at the amino acid position given under "pos" are denoted by "/" in the sequence. In topologies "xx/" denotes the signal peptidase cleavage site at position xx, "i" refers to the cytosolic face and "o" to the luminal/extracellular face of membranes. GPI anchor attachment sites are given in parentheses. The terminal number denotes the full length of the protein. Click here for file

Additional file 3 **Proteins cloned for expression in *E. coli***. A total of 43 proteins were selected for expression in *E. coli*. The expression constructs included only the mature parts of the proteins, from signal peptidase cleavage site to the omega site of the GPI-anchoring sequence. For explanation of topology nomenclature see Additional file 2. Also shown is the outcome of the expression experiments and the purification approach. Click here for file

Additional file 4 Oligonucleotides used in this study. Click here for file
