# Supplementary material for: Processing of predicted substrates of fungal Kex2 proteinases from Candida albicans, C. glabrata, Saccharomyces cerevisiae and Pichia pastoris
Source: BMC Microbiol. 2008 Jul 14;8:116. doi: 10.1186/1471-2180-8-116 (PMC2515848; doi:10.1186/1471-2180-8-116)
Supplement: Additional file 1 — Purity of Kex2 preparations. (A) Preparations of C. glabrata (Cg), S. cerevisiae (Sc) and Pichia pastoris (Pp) soluble Kex2 enzymes were pure, as shown by silver staining. (B) To ensure, that the observed proteolytic activity observed in culture supernatant of the CaKex2 expressing strains was not due to another secreted protease, the activity was monitored in parallel in the parental wild type (SC5314). The wild type did not have this activity. (C) Furthermore, the activity against the substrate CA0365 from the CaKex2 expressing strain was inhibited by PMSF, EDTA and ZnCl2, but not by Pepstatin A, an inhibitor of the secretory aspartic proteinases of C. albicans. Also shown (first lane) is the missing activity of ScKex2 against this substrate. [file 1471-2180-8-116-S1.ppt]

## Slide 1
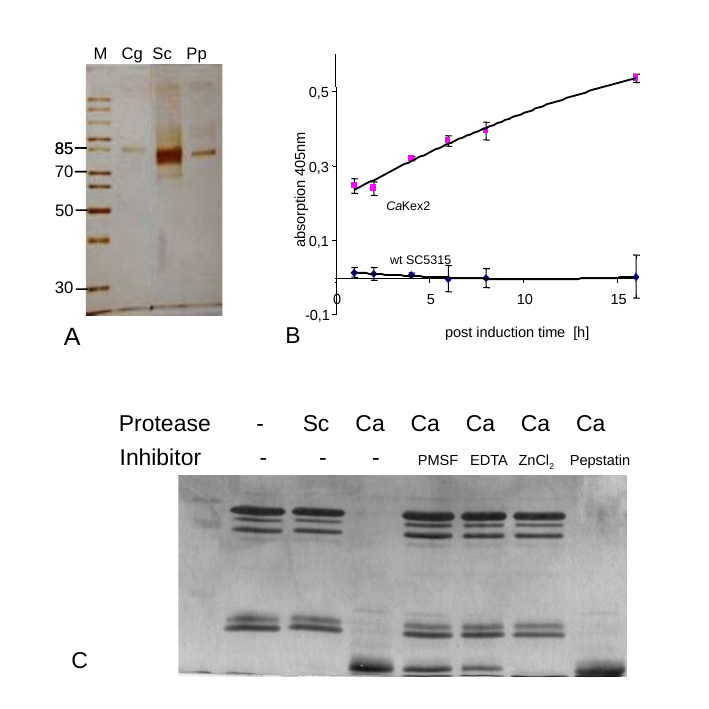

M Cg Sc Pp
0,5
0,3
absorption 405nm
Ca
Kex2
0,1
wt SC5315
0
5
10
15
-0,1
post induction time [h]
85
85
70
CaKex2-Act#7
50
30
A
B
Protease - Sc Ca Ca Ca Ca Ca
Inhibitor - - - PMSF EDTA ZnCl2 Pepstatin
C
