# Supplementary material for: Processing of predicted substrates of fungal Kex2 proteinases from Candida albicans, C. glabrata, Saccharomyces cerevisiae and Pichia pastoris
Source: BMC Microbiol. 2008 Jul 14;8:116. doi: 10.1186/1471-2180-8-116 (PMC2515848; doi:10.1186/1471-2180-8-116)
Supplement: Additional file 2 — Selected groups of predicted substrates with conserved cleavage sites. Selected groups of proteins with conserved predicted Kex2 cleavage sites are listed. Potential Kex2 cleavage sites at the amino acid position given under "pos" are denoted by "/" in the sequence. In topologies "xx/" denotes the signal peptidase cleavage site at position xx, "i" refers to the cytosolic face and "o" to the luminal/extracellular face of membranes. GPI anchor attachment sites are given in parentheses. The terminal number denotes the full length of the protein. [file 1471-2180-8-116-S2.doc]

α-mating pheromones

| species | name | pos | sequence | PSSM | Topology |
| --- | --- | --- | --- | --- | --- |
| *C.glabrata* | CAGL0H03135g | 85 | LFVNTTLYNQATKGEKLSDFTKR/DANPDAEAEA | 402 | 21/(no TMs)159 |
| *C.glabrata* | CAGL0H03135g | 110 | NPDAEAEAWHWVKIRKGQGLFRR/SADASPEAEA | 39 | 21/(no TMs)159 |
| *C.glabrata* | CAGL0H03135g | 135 | DASPEAEAWHWVRLRKGQGLFRR/SADASPEAEA | 39 | 21/(no TMs)159 |
| *S. cerevisiae* | YGL089C/ MF2 | 100 | KREAVADAWHWLNLRPGQPMYKR/EANADAWHWL | 392 | 20/(no TMs)120 |
| *S. cerevisiae* | YPL187W / MF1 | 84 | LLFINTTIASIAAKEEGVSLDKR/EAEAWHWLQL | 21 | 20/(no TMs)165 |
| *S. cerevisiae* | YPL187W / MF1 | 103 | LDKREAEAWHWLQLKPGQPMYKR/EAEAEAWHWL | 392 | 20/(no TMs)165 |
| *S. cerevisiae* | YPL187W /MF1 | 124 | KREAEAEAWHWLQLKPGQPMYKR/EADAEAWHWL | 392 | 20/(no TMs)165 |
| *S. cerevisiae* | YPL187W /MF1 | 145 | KREADAEAWHWLQLKPGQPMYKR/EADAEAWHWL | 392 | 20/(no TMs)165 |
| *C. famata* | DEHA0F20900g | 104 | GIDIEGLEAAFAKSDDTASVSKR/DANADAEAKF | 312 | 21/(no TMs)227 |
| *C. famata* | DEHA0F20900g | 129 | NADAEAKFHWMTYRFFQPNLRKR/EANADAKFHW | 47 | 21/(no TMs)227 |
| *C. famata* | DEHA0F20900g | 152 | EANADAKFHWMTYRFFQPNLKKR/EANADAEAKF | 47 | 21/(no TMs)227 |
| *C. albicans* | CA2412 / MFα1 | 80 | DNKPVILIVNGTTLTSGANNEKR/EAKSKGGFRL | 264 | 19/(no TMs)143 |
| *C. albicans* | CA2412 / MFα1 | 101 | KREAKSKGGFRLTNFGYFEPGKR/DANADAGFRL | 122 | 19/(no TMs)143 |
| *C. albicans* | CA2412 / MFα1 | 122 | KRDANADAGFRLTNFGYFEPGKR/DANAEAGFRL | 122 | 19/(no TMs)143 |

Opaque-phase specific protein Ops4 and homologs

| species | name | pos | sequence | PSSM | Topology |
| --- | --- | --- | --- | --- | --- |
| *C. albicans* | CA2974 | 54 | LTVRESNLVNSALANLQHYNAKR/DLMSQEEIIK | 273 | 21/(no TMs)328 |
| *C. albicans* | CA2974 | 65 | ALANLQHYNAKRDLMSQEEIIKR/ENQIVTDVLT | 134 | 21/(no TMs)328 |
| *C. albicans* | CA2974 | 180 | NLIGNALGLNANTVSSKMASAKR/EIMVESAPEP | 94 | 21/(no TMs)328 |
| *C. albicans* | CA3713 / Ops4 | 33 | *ANIVSEQTMVKR/EDVNAIVELI | 420 | 21/(no TMs)403 |
| *C. albicans* | CA3713 / Ops4 | 53 | VKREDVNAIVELINEIKHINQKR/DLAEGEDLLE | 303 | 21/(no TMs)403 |
| *C. albicans* | CA3713 / Ops4 | 67 | EIKHINQKRDLAEGEDLLELQRR/ADSVIGELVS | 28 | 21/(no TMs)403 |
| *C. albicans* | CA3713 / Ops4 | 191 | SSSSSSAAPAAAAAPATNGASKR/EIMEAAEYLS | 139 | 21/(no TMs)403 |
| *C. famata* | DEHA0C08173g | 33 | *AGTPTTTSLTKR/DAENIGKAIA | 60 | 21/(no TMs)329 |
| *C. famata* | DEHA0C08173g | 52 | LTKRDAENIGKAIALLNDYNAKR/ETSPSYALAE | 273 | 21/(no TMs)329 |
| *C. famata* | DEHA0C08173g | 63 | AIALLNDYNAKRETSPSYALAER/DYPIVTTVLG | 4 | 21/(no TMs)329 |
| *C. famata* | DEHA0C08173g | 170 | GSYIGDLFDKIKDKISNIGSSKR/ESLEERALTT | 94 | 21/(no TMs)329 |
| *C. famata* | DEHA0E15136g | 33 | *AAVVAENSLVTR/GDANEILEIL | 9 | 21/(no TMs)367 |
| *C. famata* | DEHA0E15136g | 53 | VTRGDANEILEILSSLKQVNEKR/DLVSESESFE | 264 | 21/(no TMs)367 |
| *C. famata* | DEHA0E15136g | 67 | SLKQVNEKRDLVSESESFELSKR/ADGLVTELVA | 50 | 21/(no TMs)367 |
| *C. famata* | DEHA0E15136g | 218 | SSGSDVDISGIPSPSEYNSGAKR/DFIDGEFLDK | 16 | 21/(no TMs)367 |
| *C. famata* | DEHA0F12496g | 73 | IHSLVSSLNQYNAAHKVPGYTKR/EDILVREAHE | 315 | 23/(no TMs)310 |
| *C. famata* | DEHA0F12496g | 187 | LPGLLRIGKELLKQNGIDIFSKR/SIDTTELETA | 334 | 23/(no TMs)310 |
| *C. famata* | DEHA0F12496g | 208 | KRSIDTTELETAAVFEETSIDKR/ESQLLNELFA | 65 | 23/(no TMs)310 |
| *C. albicans* | CA1873 | 39 | *EQQQQQESQFASKALTKR/EEQDIQELVQ | 60 | 21/(no TMs)351 |
| *C. albicans* | CA1873 | 58 | LTKREEQDIQELVQHINNYKTRR/DAIDEEIMKR | 246 | 21/(no TMs)351 |
| *C. albicans* | CA1873 | 68 | ELVQHINNYKTRRDAIDEEIMKR/DYAIVTDVLA | 173 | 21/(no TMs)351 |
| *C. albicans* | CA1873 | 195 | QLITRDETDIDALAPYVVTMEKR/LDLDGVVDNL | 286 | 21/(no TMs)351 |
| *C. albicans* | CA4679 / Pga17 | 322 | FGSVPTSINYSALSQISGALRKR/EYNDAVEAAL | 47 | 20/(no TMs)(535-GPI)557 |
| *C. albicans* | CA4679 / Pga17 | 338 | SGALRKREYNDAVEAALREIQKR/EEGIDDVEIA | 173 | 20/(no TMs)(535-GPI)557 |
| *C. albicans* | CA4679 / Pga17 | 354 | LREIQKREEGIDDVEIALRKMKR/DNIEDLLTTI | 445 | 20/(no TMs)(535-GPI)557 |
| *C. albicans* | CA4679 / Pga17 | 457 | SRAFNDDELKAVLWNDITSIFKR/DMAFRDEIVK | 241 | 20/(no TMs)(535-GPI)557 |
| *C. albicans* | CA4679 / Pga17 | 468 | VLWNDITSIFKRDMAFRDEIVKR/SNGTITSLPV | 222 | 20/(no TMs)(535-GPI)557 |

other polypeptides

| species | Name | pos | sequence | PSSM | topology |
| --- | --- | --- | --- | --- | --- |
| *C. albicans* | CA1402 /Ece1 | 30 | *IHHAPEFNMKR/DVAPAAPAAP | 303 | 19/(no TMs)271 |
| *C. albicans* | CA1402 /Ece1 | 60 | AAPADQAPTVPAPQEFNTAITKR/SIIGIIMGIL | 187 | 19/(no TMs)271 |
| *C. albicans* | CA1402 /Ece1 | 92 | LGNIPQVIQIIMSIVKAFKGNKR/EDIDSVVAGI | 18 | 19/(no TMs)271 |
| *C. albicans* | CA1402 /Ece1 | 125 | IADMPFVVRAVDTAMTSVASTKR/DGANDDVANA | 114 | 19/(no TMs)271 |
| *C. albicans* | CA1402 /Ece1 | 159 | VRLPEIVARVATGVQQSIENAKR/DGVPDVGLNL | 273 | 19/(no TMs)271 |
| *C. albicans* | CA1402 /Ece1 | 193 | ANAPRLISNVFDGVSETVQQAKR/DGLEDFLDEL | 228 | 19/(no TMs)271 |
| *C. albicans* | CA1402 /Ece1 | 227 | QRLPQLITRSAESALKDSQPVKR/DAGSVALSNL | 158 | 19/(no TMs)271 |
| *C. famata* | DEHA0E07403g | 31 | *ATYESSVQKR/ADIDQLAEAL | 346 | 21/(no TMs)348 |
| *C. famata* | DEHA0E07403g | 51 | QKRADIDQLAEALRELQSFNEKR/DTLSTHLEKR | 264 | 21/(no TMs)348 |
| *C. famata* | DEHA0E07403g | 61 | EALRELQSFNEKRDTLSTHLEKR/EYEIVTKVLA | 48 | 21/(no TMs)348 |
| *C. famata* | DEHA0E07403g | 194 | LEVIQHQKRSSSQLQPAAEFDKR/DLNDVVVNLL | 141 | 21/(no TMs)348 |
| *C. famata* | DEHA0E07403g | 180 | SLVKEGISKLSQRDLEVIQHQKR/SSSQLQPAAE | 451 | 21/(no TMs)348 |
| *C. albicans* | CA3122 | 41 | *IFDLESLKQGLQDEETVNNDKR/EPVNLLYLDR | 115 | 19/(no TMs)(?-GPI)410 |
| *C. albicans* | CA3122 | 68 | LLYLDRFKMGVSDEAKGNAKFKR/DPKNVIDPAS | 621 | 19/(no TMs)(?-GPI)410 |
| *C. albicans* | CA3122 | 92 | PKNVIDPASLKEGSAEEEQKDKR/EPKNLFNLQA | 169 | 19/(no TMs)(?-GPI)410 |
| *C. albicans* | CA3122 | 116 | PKNLFNLQALHEGLKDEETKSKR/EAKNLPNLEA | 401 | 19/(no TMs)(?-GPI)410 |
| *C. albicans* | CA3122 | 161 | KDAKNLIDLVALKQSLEKEAAKR/DAKNIPDLEA | 139 | 19/(no TMs)(?-GPI)410 |
| *C. albicans* | CA3122 | 186 | KNIPDLEALKTGIEEEEGQVAKR/DAKNVINLSN | 312 | 19/(no TMs)(?-GPI)410 |
| *C. albicans* | CA3122 | 204 | QVAKRDAKNVINLSNFIETPSKR/EGKNLFDLTK | 111 | 19/(no TMs)(?-GPI)410 |
| *C. albicans* | CA3122 | 225 | KREGKNLFDLTKFQQSGQPIKKR/DQKILKQEKS | 146 | 19/(no TMs)(?-GPI)410 |
| *C. albicans* | CA3295 | 318 | GGILGGSSGYTGSTSKVCTLKKR/SLEKKEIRKL | 47 | 23/(no TMs)(?-GPI)1249 |
| *C. albicans* | CA3295 | 354 | SVNKVIARRQQAIAEKKQANAKR/ESISFVSFEA | 273 | 23/(no TMs)(?-GPI)1249 |
| *C. albicans* | CA3295 | 401 | VTAVSSVLLTVAALTNANEIDKR/SFFGDLFSGL | 65 | 23/(no TMs)(?-GPI)1249 |
| *C. albicans* | CA3295 | 551 | APSSEAPSSSSSEALSSSSTTKR/PTAAAKGFFG | 114 | 23/(no TMs)(?-GPI)1249 |
| *S. cerevisiae* | YFL051C | 33 | *EACLPTNKR/EDGMNINFYE | 102 | 24/(no TMs)160 |
| *S. cerevisiae* | YFL051C | 99 | LSIYYSPPCESTPTCVTYAVLKR/DEDGYDPCGP | 439 | 24/(no TMs)160 |
| *S. cerevisiae* | YFL051C | 116 | YAVLKRDEDGYDPCGPLYETKKR/DTEYCDPNTA | 88 | 24/(no TMs)160 |
| *C. albicans* | CA0365 | 31 | *IPQESTTEVDKR/LDADVAAQLA | 131 | 19/(no TMs)178 |
| *C. albicans* | CA0365 | 62 | LALNILNLIQLGIGANVNVTAKR/DESAVTIEQS | 94 | 19/(no TMs)178 |
| *C. albicans* | CA0365 | 74 | IGANVNVTAKRDESAVTIEQSKR/LDADVAAQLA | 228 | 19/(no TMs)178 |
| *C. albicans* | CA0365 | 105 | LAGNILNLIQLGAGAEISASTKR/DESAVDTVEE | 114 | 19/(no TMs)178 |
| *C. albicans* | CA0365 | 118 | GAEISASTKRDESAVDTVEESKR/LDADVAAQIA | 5 | 19/(no TMs)178 |
| *C. albicans* | CA0365 | 149 | IAANILNLIQLGIGADVNVSAKR/EDTVAAQIGA | 94 | 19/(no TMs)178 |

glucanases

| species | Name | pos | sequence | PSSM | topology |
| --- | --- | --- | --- | --- | --- |
| *C. glabrata* | CAGL0I00484g / Exg1 | 38 | *VIAPKNKDTSLHFVNEKR/YYDYDSKAIG | 264 | 20/(no TMs)443 |
| *S. cerevisiae* | YLR300W / Exg1 | 39 | *VPARDPSSIQFVHEENKKR/YYDYDHGSLG | 256 | 20/(no TMs)448 |
| *C. albicans* | CA0822 / Exg1 | 37 | *ISNPFKPNGNLKFKR/GGGHNVAWDY | 621 | 22/(no TMs)438 |
| *A. gossipii* | AAR146W | 44 | *LPALTPNSIQVVTNTKR/YFDYENKTMR | 328 | 27/(no TMs)442 |
| *C. albicans* | CA3867 / Phr2 | 73 | QQNNLDSNESFVDPLANPEHCKR/DIPYLEAVDT | 451 | 23/(no TMs)(510-GPI)544 |
| *C. albicans* | CA4857 / Phr1 | 83 | SGYDADPNRKYNDPLADADACKR/DVKYFKESNT | 154 | 25/(no TMs)(515-GPI)548 |
| *C. albicans* | CA5572 / Bgl22 | 59 | TSSINHKNQPLSLRNNPQLIQKR/SRHAESPLFI | 173 | 21/(no TMs)924 |
| *Y. lipolytica* | YALI0F05390g | 85 | DANVPVDEYHYTAWLGKEEAEKR/LTDHWNTWIT | 135 | 16/(no TMs)421 |

structural cell wall proteins

| species | Name | pos | sequence | PSSM | topology |
| --- | --- | --- | --- | --- | --- |
| *C. albicans* | CA0177 | 123 | DEGSDCDDDDCKKKKKAHRYTKR/CGGGDDDDCE | 315 | 19/(no TMs)275 |
| *C. albicans* | CA0856 | 190 | SGSDCEDDECKKRKKKVHRNYKR/GGYGSERRQS | 363 | o28-45i57-78o356 |
| *C. albicans* | CA0856 | 198 | ECKKRKKKVHRNYKRGGYGSERR/QSDCESDCER | 46 | o28-45i57-78o356 |
| *C. albicans* | CA0856 | 211 | KRGGYGSERRQSDCESDCERSER/CNYPFCELYE | 50 | o28-45i57-78o356 |
| *C. albicans* | CA1334 | 116 | YRNENSEEECSDEDDDHHKKKKR/PHRHGGKSDD | 376 | 19/(no TMs)201 |
| *C. albicans* | CA1334 | 135 | KKKRPHRHGGKSDDDDDDKKWKR/GGDYSDDNDN | 552 | 19/(no TMs)201 |
| *C. albicans* | CA1844 | 123 | DEGSDCDDDDCKKKKKAHRYAKR/CGGGDDDDCE | 262 | 19/(no TMs)275 |
| *C. albicans* | CA2912 | 84 | DDGQLERNDKKLDCNCKSERVSR/PAPSPSAIAV | 31 | 19/(no TMs)247 |
| *C. albicans* | CA2912 | 119 | GNEECDEDCDDEHRKKGSKQYKR/GEVENPRETR | 303 | 19/(no TMs)247 |
| *C. albicans* | CA2912 | 129 | DEHRKKGSKQYKRGEVENPRETR/DCDFCTIEKS | 70 | 19/(no TMs)247 |
| *C. albicans* | CA3030 | 70 | IQVVTVESASALSTDTATSTLTR/NDNKKEATPV | 17 | 19/(no TMs)(328-GPI)346 |
| *C. albicans* | CA3614 | 121 | DNDECDEDCDDEDKKKGHKQYKR/GEVEEPCETS | 303 | 19/(no TMs)251 |
| *C. albicans* | CA3720 | 128 | DDDDDNCDDDCKKKKKKVYFAKR/GDDDDDDKCD | 334 | 25/(no TMs)274 |
| *C. albicans* | CA2942 / Ccw14 | 222 | VNYYSNQQASDNPKPHKVQHVKR/IKKFPKKVSK | 578 | 24/(noTMS)271 |
| *C. glabrata* | CAGL0I06160g | 60 | AKPEYTASFGIAVKPISSSVAKR/AVVSQIGDGQ | 312 | 19/(no TMs)233 |
| *C. glabrata* | CAGL0I06182g | 61 | TDYASTFGIAVQPIATPASVAKR/AVSQIGDGQV | 312 | 19/(no TMs)340 |
| *C. glabrata* | CAGL0I06204g | 61 | TDYASTFGIAVQPIATPASVAKR/AVSQIGDGQV | 312 | 19/(no TMs)349 |
| *C. glabrata* | CAGL0M08492g | 62 | TDYSSTFGIAVQPIATSSSVAKR/AVSQIGDGQV | 312 | 20/(no TMs)335 |
| *K. lactis* | KLLA0B07392g | 81 | VSTGTATSTSAKTTATTTSKAKR/DVAAISQIGD | 401 | 19/(no TMs)362 |
| *S. cerevisiae* | YJL158C / Cis3 | 63 | GAAEYTTTFGIAVQAITSSKAKR/DVISQIGDGQ | 401 | 22/(no TMs)(209-GPI)227 |
| *S. cerevisiae* | YJL160C | 61 | TEYRTSFGLAVIPFTVTESKVKR/NVISQINDGQ | 571 | 24/(no TMs)180 |
| *S. cerevisiae* | YKL163W / Pir3 | 66 | SFGIAIEAVATSASSVASSKAKR/AASQIGDGQV | 401 | 19/(no TMs)325 |
| *S. cerevisiae* | YKL164C / Pir1 | 62 | DYSSTFGIAVEPIATTASSKAKR/AAAISQIGDG | 401 | 19/(no TMs)341 |

Pry-proteins

| species | Name | pos | sequence | PSSM | topology |
| --- | --- | --- | --- | --- | --- |
| *A. gossipii* | AAL179W | 233 | RPAGGQNGASFEEEILRAHNSRR/QLHKDTKPLT | 139 | 20/(no TMs)360 |
| *C. albicans* | CA0229 | 193 | GTNDDSQLSSFSRQILEAHNIKR/ASHGVNPLTW | 234 | 24/(no TMs)317 |
| *C. albicans* | CA5344 | 134 | SDATPTADVEFAEEILKEHNVKR/ALHGVPALSW | 388 | 20/(no TMs)271 |
| *K. lactis* | KLLA0D02442g | 242 | SSSSDSNLSSFASSMLDEHNAKR/ALHKDTPTLS | 273 | i12-39o45-63i84-102o368 |
| *S. cerevisiae* | YJL079C / Pry1 | 173 | ASSSDSDLSDFASSVLAEHNKKR/ALHKDTPALS | 256 | 20/(no TMs)299 |
| *S. cerevisiae* | YKR013W / Pry2 | 203 | ASSTQSSSSDFSTSMVNEHNTKR/ALHKDTGSLT | 328 | 19/(no TMs)329 |
| *C. glabrata* | CAGL0F05137g | 76 | EKKTKTKSQKKTKPTPSTDLDKR/AQKKRSNLSE | 21 | 18/(no TMs)227 |
| *C. glabrata* | CAGL0F05137g | 80 | TKSQKKTKPTPSTDLDKRAQKKR/SNLSEWQQKM | 256 | 18/(no TMs)227 |
| *C. glabrata* | CAGL0F05137g | 98 | AQKKRSNLSEWQQKMLDQHNKKR/ELHKDTDSLV | 214 | 18/(no TMs)227 |
| *C. glabrata* | CAGL0G07667g | 132 | SPAQDSNLSDFAKSMLNEHNIKR/ALHQDTNPLT | 234 | 18/(no TMs)258 |

aspartic proteinases

| species | Name | pos | sequence | PSSM | topology |
| --- | --- | --- | --- | --- | --- |
| *C. albicans* | CA2660 / Sap1 | 49 | VTLDFDVIKTPVNATGQEGKVKR/QALPVTLNNE | 571 | 24/(no TMs)391 |
| *C. albicans* | CA3138 / Sap2 | 55 | FSVVKTPKAFPVTNGQEGKTSKR/QAVPVTLHNE | 94 | 19/(no TMs)398 |
| *C. albicans* | CA6065 / Sap3 | 57 | VIKTHKNVTGPQGEINTNVNVKR/QTVPVKLINE | 388 | 19/(no TMs)398 |
| *C. albicans* | CA2055 / Sap4 | 22 | *VKR/STGFVTLDFN | N/A | 19/(no TMs)417 |
| *C. albicans* | CA2055 / Sap4 | 35 | *VKRSTGFVTLDFNVKR/SLVDPKDPTV | 388 | 19/(no TMs)417 |
| *C. albicans* | CA2055 / Sap4 | 74 | LFLDIEPTEIPVDDTGRNDVGKR/GPVAVKLDNE | 341 | 19/(no TMs)417 |
| *C. albicans* | CA2499 / Sap5 | 35 | *GFVTLDFNVKR/SLVDPDDPTV | 388 | 24/(no TMs)418 |
| *C. albicans* | CA2499 / Sap5 | 75 | FLEFTPSEFPVDETGRDGDVDKR/GPVAVTLHNE | 131 | 24/(no TMs)418 |
| *C. albicans* | CA0968 / Sap6 | 35 | *GFVTLDFNVKR/SLVDPDDPTV | 388 | 24/(no TMs)418 |
| *C. albicans* | CA0968 / Sap6 | 75 | FLDLDPTKIPVDDTGRNDGVDKR/GPVAVKLDNE | 131 | 24/(no TMs)418 |
| *C. albicans* | CA1266 / Sap8 | 32 | *EDIDKR/AEKVVSLDFT | 65 | 26/(no TMs)405 |
| *C. albicans* | CA1266 / Sap8 | 74 | AHGQHHQSQQQQQQQQQQPAQKR/GTVQTSLINE | 154 | 26/(no TMs)405 |
| *C. albicans* | CA4700 / Sap9 | 49 | VRRGESKDDLSPEDDSNPRFVKR/DGSLDMTLTN | 475 | 18/(no TMs)(520-GPI)544 |
| *C. albicans* | CA4863 / Sap10 | 37 | *KLDFNKVSTPSKYTKR/DALPMPLIND | 315 | 21/(no TMs)(428-GPI)453 |
| *C. famata* | DEHA0A03729g | 51 | DFDVYQGLNLSAALSSYSQISRR/DGSYDVSLVN | 79 | 23/(no TMs)429 |
| *C. glabrata* | CAGL0E01419g | 49 | YCKNSDNKSRNLDALGSNRFAKR/ASSVASASPS | 334 | ?/(noTMs)(?-GPI)519 |
| *C. glabrata* | CAGL0E01727g | 30 | *DFERQTAQDVALAKR/HTSNGVANAM | 50 | 15/(no TMs)(519-GPI)539 |
| *C. glabrata* | CAGL0E01749g | 33 | *KLDFEKVPEQNLALNKR/DDKGLQLELE | 54 | 16/(no TMs)482 |
| *C. glabrata* | CAGL0E01771g | 33 | *DFEKTPKISDELTKR/ADNDSFIAPL | 60 | 18/(no TMs)(501-GPI)519 |
| *C. glabrata* | CAGL0E01793g | 32 | *KLDFTRVPSASVLEKR/ADDLSPVPLR | 48 | 16/(no TMs)(480-GPI)516 |
| *C. glabrata* | CAGL0E01815g | 33 | *KLDFEKSPKASSDLVKR/DDEYVNVPLK | 71 | 16/(no TMs)(503-GPI)519 |
| *C. glabrata* | CAGL0E01837g | 32 | *KLDFTKTPGSDLAKR/DVVDPEAAQL | 50 | 17/(no TMs)(498-GPI)521 |
| *C. glabrata* | CAGL0E01859g | 29 | *ALDFEKSHGDDLVKR/DVQDVKLINS | 71 | 14/(no TMs)(484-GPI)505 |
| *C. glabrata* | CAGL0E01881g | 30 | TAAAAGYLRLPFTKITDNSLAKR/ADDDYLNVQL | 50 | (no TMs)(469-GPI)508 |
| *C. glabrata* | CAGL0J02288g | 37 | VVNCLRLTIEKRIVSSHASLSKR/SAVDLQFRRF | 50 | (no TMs)(508-GPI)541 |
| *C. glabrata* | CAGL0J02288g | 46 | EKRIVSSHASLSKRSAVDLQFRR/FNNLYYESVL | 180 | (no TMs)(508-GPI)541 |
| *C. glabrata* | CAGL0M04191g | 44 | ADLSYVKLDFDKYYGETFETAKR/GRSQADIRVN | 94 | 19/(no TMs)(580-GPI)601 |
| *C. glabrata* | CAGL0M04191g | 56 | YYGETFETAKRGRSQADIRVNKR/ANGYEEVQIT | 336 | 19/(no TMs)(580-GPI)601 |
| *K. lactis* | KLLA0D15917g | 44 | FDHDSKVIQLQLLKPADRSLQKR/SKSFESKLNP | 55 | 19/(no TMs)(489-GPI)511 |
| *K. lactis* | KLLA0E03938g | 23 | *ITKR/DVSQVGEANK | 187 | 19/(no TMs)(556-GPI)589 |
| *K. lactis* | KLLA0E03938g | 64 | KLRGNDASEASLSKRRVGHLKKR/ADGYVDVEID | 47 | 19/(no TMs)(556-GPI)589 |
| *S. cerevisiae* | YLR120C / Yps1 | 29 | *IPAANKR/DDDSNSKFVK | 150 | 22/(no TMs)(542-GPI)569 |
| *S. cerevisiae* | YDR144C / Yps2 | 64 | KKYGSSFENALDDTKGRTRLMTR/DDDYELVELT | 7 | 23/(no TMs)(575-GPI)596 |
| *S. cerevisiae* | YLR121C / Yps3 | 46 | DGKYVKIPFTKKKNGDNGELSKR/SNGHEKFVLA | 50 | 21/(no TMs)(483-GPI)508 |
| *S. cerevisiae* | YGL259W / Yps5 | 51 | FPVQKFADIINIGTQDVSTVFKR/NEVLNTTVIN | 483 | 22/(no TMs)165 |
| *S. cerevisiae* | YIR039C / Yps6 | 51 | FPVQKLADIINICTQDVSTVFKR/NEVLNTTVIN | 483 | 22/(no TMs)537 |

proteins with His-box

| Sun family | | pos | sequence | PSSM | topology |
| --- | --- | --- | --- | --- | --- |
| *T. reesei* | Tr46210 | 39 | *PSHHHHHAHRHAKKHAAARVEKR/APDVVTEVV | 302 | 17/(no TMs)436 |
| *S. nodorum* | SNU10592.1 | 33 | *RHAHQVVKR/DARPDAVVY | 444 | 25/(no TMs)467 |
| *A. gossipii* | ADR322W | 27 | *PTHKHRRHRR/DSQGHLTMT | 444 | 18/(no TMs)508 |
| *A. gossipii* | AER372C | 40 | *EDCSTSAGHQHKR/AIAVEYVYE | 357 | 28/(no TMs)436 |
| *A. fumigatus* | Afu7g05450 | 31 | *PHHHQHRHHQHKR/EDVVESSAT | 357 | 20/(no TMs)414 |
| *A. nidulans* | AN6697.2 | 31 | *QHQHGHHHQHSKR/EVVTVDGPT | 407 | 19/(no TMs)410 |
| *C. albicans* | CA0883 | 45 | *DNKNIKR/EDCDKTSFHGHHKHKR/AVAYDYAYV | 627 | 23/(no TMs)418 |
| *C. albicans* | CA5232 | 30 | *PLQHQHHHHHEHARR/AEVTKVVYV | 207 | 16/(no TMs)372 |
| *C. glabrata* | CAGL0G08668g | 44 | *PQFDRYKEHSKKDVSHQHQRKR/AVTVEYVYA | 214 | 23/(no Tms)(449-GPI)469 |
| *C. glabrata* | CAGL0J09922g | 42 | *AMPDLKDCTTTAQGNHQHKR/AVAVEYVYE | 357 | 23/(no TMs)427 |
| *C. glabrata* | CAGL0L03289g | 29 | *PAVQHQDKHAHEKR/DLVVVTEYV | 394 | 16/(no TMs)366 |
| *C. glabrata* | CAGL0L05434g | 30 | *PSPKHDHEDRHAIKR/DVDVVTVVE | 119 | 16/(no Tms)(326-GPI)346 |
| *D. hansenii* | DEHA0C15092g | 31 | *PLEGHKHHQHQHKR/DSVVKQVTQ | 357 | 18/(no TMs)384 |
| *D. hansenii* | DEHA0F22242g | 42 | *PAPAQDEDCATTQVHAHHKHKR/EVVYDYAYV | 627 | 21/(no TMs)404 |
| *F. graminearum* | FG01351.1 | 40 | *HHNHQHIHARRHAGSKVEKR/SPDVITEYV | 207 | 20/(no TMs)443 |
| *K. lactis* | KLLA0E14982g | 33 | *PAPGSKHIHRKEKR/AAVTVTQYY | 389 | 20/(no TMs)398 |
| *K. lactis* | KLLA0F04433g | 43 | *PHAHAKR/DEDCSTTVHAHHKHKR/AVAVEYVYQ | 627 | 21/(no TMs)413 |
| *M. grisae* | MG00505.4 | 37 | *HANHHRHLHAKRDVAKR/SPEVVVKYE | 312 | 21/(no TMs)416 |
| *N. crassa* | NCU02668.1 | 39 | *PHNHHGHHHAKKHAHAGIDKR/ADGTVVVTE | 65 | 19/(no TMs)445 |
| *S. pombe* | SPAC1002.13c | 30 | *PFRHPHHLLNKR/DVSVVTSKV | 54 | 19/(no TMs)417 |
| *S. pombe* | SPBC2G2.17c | ? | *PLGNAPYHHHHHAGL.NASNITVGV | 0 | 20/(no TMs)319 |
| *Y. lipolytica* | YALI0E33539g | 42 | *PATPDADCNEEKEVR/DFHAHHQHKR/AVQVEYVYV | 357 | 18/(no Tms)(377-GPI)414 |
| *S. cerevisiae* | YIL123W | 34 | *PHVDVHQEDAHQHKR/AVAYKYVYE | 357 | 20/(no TMs)475 |
| *S. cerevisiae* | YJL116C | ? | *PAPAPADSHHEDHHKDEKP.AVVTVTQYI | 0 | 19/(no Tms)(318-GPI)337 |
| *S. cerevisiae* | YKR042W | 37 | *DFKPQFTLLSSVTKKKKKKVR/PHNFQCIHS | 3 | 17/(no Tms)365 |
| *S. cerevisiae* | YNL066W | 44 | *PYAADIDTGCTTTAHGSHQHKR/AVAVTYVYE | 357 | 23/(no TMs)420 |

| Scw4p/10p-like | | pos | sequence | PSSM | topology |
| --- | --- | --- | --- | --- | --- |
| *A. fumigatus* | AFU6g12380 | 25 | *HPHVGHRR/NAEHDVKPK | 13 | 18/(no TMs)369 |
| *A. gossipii* | AGL354C | 28 | *EVAHEHHDRR/AVAYVTNYR | 87 | 19/(no TMs)451 |
| *C. albicans* | CA6263 | 31 | *APLAHQHHQHKEEKR/AVHVVTTTN | 5 | 17/(no Tms)??? |
| *C. glabrata* | CAGL0G00308g | 28 | *PHAAHTHKEKR/DVVTKTMHA | 389 | 18/(no TMs)374 |
| *C. glabrata* | CAGL0M13805g | 30 | *PANVHHEHKNKR/DVVTKTVHN | 432 | 19/(no TMs)371 |
| *D. hansenii* | DEHA0C13937g | ? | *PLQQHQHHEHKMEKK/DVKVVTQTS | 0 | 18/(no TMs)372 |
| *K. lactis* | KLLA0A03201g | 31 | *AAAVHKHHAAEEKR/DVTVVVTNF | 125 | 18/(no TMs)378 |
| *K. lactis* | KLLA0C14047g | 28 | *PIHGHKDNKR/DLVYVTNRV | 6 | 19/(no TMs)378 |
| *Y. lipolytica* | YALI0D20680g | 29 | *PIVGHHAHHQHKR/EAGIETVFV | 357 | 17/(no TMs)420 |
| *S. cerevisiae* | YGR279C | 29 | *PANHEHKDKR/AVVTTTVQK | 169 | 20/(no TMs)386 |
| *S. cerevisiae* | YMR305C | 28 | *PAVRHKHEKR/DVVTATVHA | 394 | 19/(no TMs)389 |

| Tos1p-like | | pos | sequence | PSSM | topology |
| --- | --- | --- | --- | --- | --- |
| *A. gossipii* | ADR056W | 116 / 125 | GSDGGSEQKP.GHKHNSGHKHKPGHDHDKR/AVHYVHVTS | 0 / 171 | 20/(no TMs)501 |
| *C. albicans* | CA2303 | 105 / 123 | YYPSSSGNSKR/QIDDQDCNVKHVHHKHKR/ATEVVQVTQ | 273 / 627 | 22/(no TMs)468 |
| *C. glabrata* | CAGL0M05599g | 100 / 120 | YPASSNSKR/AVAEEDCDSVVAKPVHKHKR/DVAVEVIEV | 273 / 627 | 18/(no TMs)446 |
| *D. hansenii* | DEHA0D16599g | 94 / 111 | YYPSSGSNNKKR/EDEEECTTKHVHHKHKR/ATEVVEVTQ | 256 / 627 | 21/(no TMs)435 |
| *F. graminearum* | FG00876.1 | 106 / 130 | SKNKR/DTESAPSPHLHGHRHLHEQRKKKR/AEWVTATID | 432 / 376 | 20/(no TMs)440 |
| *K. lactis* | KLLA0B09746g | 103 / 126 | VSFKKR/DQVAEDAPCTTVSTAKKHHEHKR/EAAVQWVEV | 313 / 8 | 19/(no TMs)457 |
| *S. pombe* | SPBP23A10.11c | 111 / 125 | YYPDTSNALSSLRKR/SNNQHMKRHPHHKR/DDVIDSTLT | 47 / 1074 | 23/(no TMs)507 |
| *Y. lipolytica* | YALI0A17919g | 115 / 141 | AKR/DAVEEEDKDCGETAKREFHAHHAHKR/GVVTEIVQV | 273 / 217 | 31/(no TMs)467 |
| *S. cerevisiae* | YBR162C | 109 / 134 | LKKR/SEKQSIESCKEGEAVVSRHKHQHKR/DVAVEYVQV | 47 / 357 | 24/(no TMs)455 |
