# Supplementary material for: Processing of predicted substrates of fungal Kex2 proteinases from Candida albicans, C. glabrata, Saccharomyces cerevisiae and Pichia pastoris
Source: BMC Microbiol. 2008 Jul 14;8:116. doi: 10.1186/1471-2180-8-116 (PMC2515848; doi:10.1186/1471-2180-8-116)
Supplement: Additional file 3 — Proteins cloned for expression in E. coli. A total of 43 proteins were selected for expression in E. coli. The expression constructs included only the mature parts of the proteins, from signal peptidase cleavage site to the omega site of the GPI-anchoring sequence. For explanation of topology nomenclature see Additional file 2. Also shown is the outcome of the expression experiments and the purification approach. [file 1471-2180-8-116-S3.doc]

Oligonucleotides for successfully used substrate expression constructs

| substrate name | forward primer | reverse primer |
| --- | --- | --- |
| CA0365 | CACCGCTATTCCACAAGAATCAACCACC | TTACAAGTCAACAGTGGTTTGAGC |
| CaEce1 | CACCGCCATCATCCACCATGCTCCAGAA | TTAAGCTTTTCCGAAATATTCTTC |
| CA1873 | CACCGCTCCAGAACAACAACAACAACAA | TTAATAGTTGGATCTTCTTCTTCT |
| CA2974 | CACCGCTGCTCCAGCTGCTGACGCTCAA | TTAGTAGTTTCTTCTCTTCTTCTT |
| CaTos1 | CACCTCATTCGAAGGCGGAAACTACTACTG | TTAGTAACCAATAGTGGCAACAGAACCAGC |
| CaSun41 | CACCAATAAAAACATCAAAAGAGAAGATTG | TTAATTATACAAGACAAAGTCAGCTTCACC |
| CaCcw14 | CACCGCTTGTTTTTTAAGTTGTATTAATGA | TTATATTGATTTTCTATTTACTTTGAATTT |
| CaPho11 | CACCGATGTTGCTGCCCCACATCAAGCATC | TTACTGATCTATTAACGGCGCATTATACTT |
| CaPga17 | CACCGTCACACCAGCATCTGATCCTTTAGC | TTAAGCATTGGAAGTTATAAAGGAATGTGA |
| CaRbt4 | CACCGCCTATGTCACCCAGACTCGTGGTGT | TTATTGTGGTCTCAAGACATTTTCAGCCAT |
| CaPir1 | CACCATTCGGTTTTCGTTAGGTGCCCAGGT | TTACCACACCTGTTGCAACACCACAATATA |
| CaCrh1 | CACCTGTAACCCATTAAAGTCAAGTGATTG | TTATTCAGTAGTCAGAACAGAAGGTTTTTC |
| CA1394 | CACCATACAAAAAGATTCACTTGGACTTAA | TTATTTAGAAAATTTTGAAATTGCATAAAA |
| CgPlb | CACCTGGTCCCCAACCAACAGTTATGTG | TTAGATCAATTGGAAAACAGCAGTCATTGC |
| CgScw4 | CACCGCTAATGTTCACCATGAACATAGG | TTAATAGATACCCCAGTACTTTTCACA |
| CAGL0L05434g | CACCCCTAAACATGACCATGAGGACAGA | TTAATTTTCGTAGACACACGTACCGCCGAC |
| CAGL0H08910g | CACCTTTATAGTACCTCAAATGAACCAT | TTAAACATGAGAAGTCGTTGTATTGCCTGG |
| CgPir1 | CACCTACGTTCCAGGTAACCCATGGTCC | TTAACACTTAACCAAGTCAATGGCAGAC |
| CAGL0A02277g | CACCTTCGACGCTTCCTCTCCAGGACGT | TCAAATGTTTCTGAAGATTTGACCCAAAC |
| CgPry1 | CACCGTTACCGTCACTAAAGAAGCACCA | TTACTTCAATGGTTTAACATTGTCGGCG |
| CgPry2 | CACCGTCACTGTCACCGAAGTTGCTCAC | TTATTTCAAAGGAGCAACATTTTGTGC |
| ScMFalpha | CACCGCTCCAGTCAACACTACAACAGAA | TTAGTACATTGGTTGGCCGGGTTTTAACTG |
| CaMFalpha | CACCGCTCCTGCTCAATACACTGGTCAAG | TTATTTACCAGGTTCAAAATAACC |
| CAGL0H03135g (MFalpha) | CACCCAGCCAGTTGGAGAAGAATTAGGCG | TTAAAATAAACCTTGGCCTTTTCTCAA |

Oligonucleotides used for Kex2 expression constructs

| species | forward primer | reverse primer |
| --- | --- | --- |
| *C. albicans* | CTATGTTGCCAATAAAATTACTAATA | GGGGCTAGCTTACAGAATTGGCCTTTTTGTTGCC |
| *C. glabrata* | CGGATCCGTATGAAGTGGTGGAACAAG | CCCGCGGCCGCTTAATGATGATGATGATGATGTATCAAATGAACACCAGTTGG |
| *P. pastoris* | CCGTACGTACCACCATG**G**ATTTGCCAGCACTTCGCTTAG | GCCGCGGCCGCTCAATGATGATGATGATGATGAGTTAGAATGCTTGTAGGAGC |

Oligonucleotides used for GST-GFP constructs

| insert | forward primer | reverse primer |
| --- | --- | --- |
| *GFP* | GGGAATTC tctaaaggtgaagaatta | GCGATGCGGCCGCTC tatttgtacaattcatcc |
| CaCcw14 | CCGGATCC aattattattccaatcaacagg | CCGAATTC tgattcattagcatttttagaa |
| CA0365 | CCGGATCC gctattccacaagaatcaacca | CCGAATTC taaggttcaaaatgttaagagc |
