# Supplementary material for: Processing of predicted substrates of fungal Kex2 proteinases from Candida albicans, C. glabrata, Saccharomyces cerevisiae and Pichia pastoris
Source: BMC Microbiol. 2008 Jul 14;8:116. doi: 10.1186/1471-2180-8-116 (PMC2515848; doi:10.1186/1471-2180-8-116)
Supplement: Additional file 4 — Oligonucleotides used in this study. [file 1471-2180-8-116-S4.doc]

| # | organism | sequence ID | kDa | predicted topology | purification condition |
| --- | --- | --- | --- | --- | --- |
| 01 | *C. albicans* | CA0365 | 21 | 19/(no TMs)806 | soluble |
| 02 | *C. albicans* | CA1402 / Ece1 | 31 | 19/(no TMs)271 | soluble |
| 04 | *C. albicans* | CA1873 / Ops4-like 1 | 39 | 21/(no TMs)351 | soluble |
| 05 | *C. albicans* | CA2412 / MFalpha | 18 | 19/(no TMs)143 | soluble |
| 06 | *C. albicans* | CA2974 / Ops4-like 2 | 37 | 21/(no TMs)328 | soluble |
| 07 | *C. albicans* | CA3713 / Ops4 | 44 | 21/(no TMs)403 | not expressed |
| 08 | *C. albicans* | CA3943 | 48 | o24-46i193 | not expressed |
| 09 | *C. albicans* | CA5814 / subtilisin | 51 | i21-40o421 | not expressed |
| 10 | *C. albicans* | CA2303 / Tos1 | 51 | 22/(no TMs)468 | refolded from IB |
| 11 | *C. albicans* | CA0883 / Sun41 | 45 | 23/(no TMs)418 | refolded from IB |
| 12 | *C. albicans* | CA2942 / Ccw14 | 33 | 17/(no TMs)271 | refolded from IB |
| 13 | *C. albicans* | CA5147 / Pho114 | 54 | 24/(no TMs)456 | refolded from IB |
| 14 | *C. albicans* | CA1583 / Rot1 | 28 | 25/(no TMs)(244-GPI)o260 | not expressed |
| 15 | *C. albicans* | CA0212 | 29 | (no TMs)226 | not expressed |
| 16 | *C. albicans* | CA2470 / Sap9 | 58 | 26/(no TMs)641 | degraded |
| 17 | *S. cerevisiae* | YDR144c / Yps3 | 63 | 23/(no TMs)(575-GPI)o596 | not expressed |
| 18 | *C. albicans* | CA3867 / Phr1 | 56 | 23/(no TMs)(510-GPI)o544 | not expressed |
| 19 | *S. cerevisiae* | YMR307w / Gas1 | 56 | 23/(no TMs)(523-GPI)o559 | not expressed |
| 20 | *C. albicans* | CA4679 / Pga17 | 58 | 20/(no TMs)(535-GPI)o557 | soluble |
| 21 | *C. albicans* | CA0104 / Rbt4 | 39 | 20/(no TMs)358 | refolded from IB |
| 22 | *C. albicans* | CA0811 / Pir-like | 39 | 19/(no TMs)177 | refolded from IB |
| 23 | *C. albicans* | CA0375 / Crh1 | 21 | 22/(no TMs)(430-GPI)o453 | soluble |
| 24 | *C. albicans* | CA2825 / Hwp1 | 65 | 20/(no TMs)(604-GPI)o634 | not expressed |
| 25 | *C. albicans* | CA6006 | 18 | 20/(no TMs)(127-GPI)o163 | not expressed |
| 26 | *C. albicans* | CA1621 / Iff6 | 103 | 20/(no TMs)(1063-GPI)o1086 | not expressed |
| 27 | *C. albicans* | CA1394 | 49 | 21/(no TMs)418 | soluble |
| 28 | *C. albicans* | CA0416 | 25 | 25/(no TMs)207 | not expressed |
| 29 | *C. albicans* | CA0915 / Kar2 | 76 | 35/(no TMs)687 | not expressed |
| 30 | *C. glabrata* | CAGL0H03135g / MFα | 19 | 21/(no TMs)159 | soluble |
| 31 | *C. glabrata* | CAGL0J11770g / Plb | 73 | 23/(no TMs)(633-GPI)o659 | refolded from IB |
| 32 | *C. glabrata* | CAGL0M13805g / Scw4 | 40 | 19/(no TMs)371 | soluble |
| 33 | *C. glabrata* | CAGL0L05434g / Sun4 | 38 | 16/(no TMs)(326-GPI)o346 | soluble |
| 34 | *C. glabrata* | CAGL0H08910g | 41 | 19/(no TMs)346 | refolded from IB |
| 35 | *C. glabrata* | CAGL0I04092g | 65 | 21/(no TMs)556 | not expressed |
| 36 | *C. glabrata* | CAGL0D02530g / Egt1 | 70 | 23/(no TMs)(656-GPI)o676 | refolded from IB |
| 37 | *C. glabrata* | CAGL0L02607g | 68 | 16/(no TMs)585 | not expressed |
| 38 | *C. glabrata* | CAGL0M08492g / Pir1 | 36 | 20/(no TMs)335 | soluble |
| 39 | *C. glabrata* | CAGL0A02277g | 31 | 24/(no TMs)272 | refolded from IB |
| 40 | *C. glabrata* | CAGL0F05137g / Pry1 | 27 | 18/(no TMs)227 | refolded from IB |
| 41 | *C. glabrata* | CAGL0G07667g / Pry2 | 29 | 18/(no TMs)258 | refolded from IB |
| 42 | *C. glabrata* | CAGL0L10802g / subtilisin | 59 | 17/(no TMs)517 | not expressed |
| 43 | *S. cerevisiae* | YPL187W/MFalpha | 20 | 20/(no TMs)165 | soluble |
